# Treatment Tone Spacing and Acute Effects of Acoustic Coordinated Reset Stimulation in Tinnitus Patients

**DOI:** 10.3389/fnetp.2021.734344

**Published:** 2021-10-06

**Authors:** Tina Munjal, Alexander N. Silchenko, Kristina J. Pfeifer, Summer S. Han, Jessica K. Yankulova, Matthew B. Fitzgerald, Ilya Adamchic, Peter A. Tass

**Affiliations:** ^1^ Department of Otolaryngology—Head and Neck Surgery, Stanford University, Stanford, CA, United States; ^2^ Department of Neurosurgery, Stanford University, Stanford, CA, United States; ^3^ Institute of Neuroscience and Medicine (INM-7: Brain and Behavior), Jülich Research Center, Jülich, Germany; ^4^ Quantitative Sciences Unit, Stanford University School of Medicine, Stanford, CA, United States; ^5^ Department of Radiology, Klinikum Friedrichshain, Berlin, Germany

**Keywords:** coordinated reset, tinnitus, auditory filter, ERB, gap index, sensorineural hearing loss (SNHL), neuromodulation, neuroplasticity

## Abstract

Acoustic coordinated reset (aCR) therapy for tinnitus aims to desynchronize neuronal populations in the auditory cortex that exhibit pathologically increased coincident firing. The original therapeutic paradigm involves fixed spacing of four low-intensity tones centered around the frequency of a tone matching the tinnitus pitch, *f*_*T*_, but it is unknown whether these tones are optimally spaced for induction of desynchronization. Computational and animal studies suggest that stimulus amplitude, and relatedly, spatial stimulation profiles, of coordinated reset pulses can have a major impact on the degree of desynchronization achievable. In this study, we transform the tone spacing of aCR into a scale that takes into account the frequency selectivity of the auditory system at each therapeutic tone’s center frequency via a measure called the gap index. Higher gap indices are indicative of more loosely spaced aCR tones. The gap index was found to be a significant predictor of symptomatic improvement, with larger gap indices, i.e., more loosely spaced aCR tones, resulting in reduction of tinnitus loudness and annoyance scores in the acute stimulation setting. A notable limitation of this study is the intimate relationship of hearing impairment with the gap index. Particularly, the shape of the audiogram in the vicinity of the tinnitus frequency can have a major impact on tone spacing. However, based on our findings we suggest hypotheses-based experimental protocols that may help to disentangle the impact of hearing loss and tone spacing on clinical outcome, to assess the electrophysiologic correlates of clinical improvement, and to elucidate the effects following chronic rather than acute stimulation.

## Introduction

Network dynamics—occurring at multiple levels and spatio-temporal scales—play a crucial role in several physiologic and pathophysiologic domains (Bashan et al., 2012; Ivanov et al., 2016). Chronic tinnitus is such a network phenomenon, across both auditory and non-auditory brain areas (Schlee et al., 2008). An EEG study revealed that acoustic coordinated reset (aCR) induced spread of desynchronization of tinnitus-related abnormal neuronal synchrony from auditory to non-auditory brain areas (Silchenko et al., 2013; Adamchic et al., 2014b). Optimizing the spread of desynchronization effects, e.g., by selecting optimal parameters of aCR stimulation, may be key to further improving therapeutic outcome. In general, a number of studies have dealt with the spreading dynamics on complex brain dynamics (O’Dea et al., 2013; Misic et al., 2015). Stimuli of different frequency (i.e., pitch) may be fed into the complex tinnitus-related brain network in a multitude of different ways, e.g., with stimulus frequencies being narrowly or widely spaced, confined to only one auditory perceptual channel or widely spread across several channels. We here study a first and fundamental step in manipulating the complex network of auditory and non-auditory brain areas by analyzing the impact of the spacing of aCR stimulation tones on acute therapeutic effects of aCR stimulation.

Tinnitus, or the perception of sound in the absence of any external stimuli (Henry et al., 2005), affects approximately 10% of the adult population (Bhatt et al., 2016). Primary tinnitus is idiopathic and may or may not be associated with sensorineural hearing loss (SNHL). Secondary tinnitus is associated with a specific underlying cause other than SNHL or an identifiable organic condition (Tunkel et al., 2014). There are three proposed mechanisms for the generation of primary tinnitus: increased neural synchrony, reorganization of tonotopic maps, and increased spontaneous firing rates (Eggermont and Tass, 2015). These mechanisms are hypothesized to be an attempt by the central nervous system (CNS) to restore evoked neural activity to pre-hearing loss levels. Nevertheless, only approximately 30% of individuals with hearing loss have tinnitus (Nondahl et al., 2011). Thus, it stands to reason that there are some purely CNS-driven factors that contribute to its generation. However, map reorganization seems not to be strictly required for the generation of tinnitus (Langers et al., 2012), and reorganization of cortical maps takes weeks, whereas abnormal synchrony can be seen rapidly following initial insult (Ortmann et al., 2011). Thus, pathological neuronal synchrony is the mechanism targeted in this study.

Abnormal neuronal synchrony has also been demonstrated to play an important role in a number of other brain disorders, including Parkinson’s disease (PD) (Lenz et al., 1994; Nini et al., 1995; Hammond et al., 2007), migraine (Angelini et al., 2004; Bjork and Sand, 2008), and epilepsy (Wong et al., 1986). To specifically counteract abnormal neuronal synchrony, coordinated reset (CR) stimulation was computationally developed based on methods from non-linear dynamics and statistical physics (Tass, 2003). CR stimulation employs sequences of phase resetting stimuli administered to neuronal sub-populations involved in abnormal synchronization processes (Tass, 2003). As shown in computational studies, in the presence of spike-timing-dependent plasticity (STDP) (Gerstner et al., 1996; Markram et al., 1997; Bi and Poo, 1998), CR stimulation may cause long-lasting desynchronization (Tass and Majtanik, 2006; Hauptmann and Tass, 2007; Popovych and Tass, 2012; Kromer and Tass, 2020). CR stimulation may reduce the rate of coincidences, which, mediated by STDP, causes a reduction of synaptic weights, ultimately shifting the network from an attractor with abnormal synaptic connectivity and abnormal neuronal synchrony to an attractor with weak connectivity and synchrony. This long-term desynchronization mechanism was coined anti-kindling (Tass and Majtanik, 2006).

The CR approach was initially developed for the treatment of PD, essential tremor, and epilepsy (Tass, 2003; Tass and Majtanik, 2006). Sustained, long-lasting (i.e., resistant to tolerance) after-effects on motor function lasting for several weeks were demonstrated in parkinsonian nonhuman primates treated with electrical CR stimulation administered to the subthalamic nucleus (STN) for a few hours only (Tass et al., 2012b; Wang et al., 2016). In contrast, effects of standard deep brain stimulation (i.e., periodic stimulation at rates greater than 100 Hz) vanished within 30 min (Tass et al., 2012b; Wang et al., 2016). Analogously, cumulative and lasting after-effects of electrical CR stimulation of the STN on Unified Parkinson’s Disease Rating Scale (UPDRS) motor scores and beta band local field power were also demonstrated in PD patients (Adamchic et al., 2014a).

As shown computationally, anti-kindling can robustly be induced in networks with STDP regardless of whether CR stimulation is administered directly to the soma or through synapses (Popovych and Tass, 2012). Subsequently, non-invasive CR was developed to treat tinnitus with acoustic stimuli (Tass et al., 2012a) and PD with vibratory stimuli (Tass, 2017; Syrkin-Nikolau et al., 2018; Pfeifer et al., 2021). Non-invasive aCR makes use of the tonotopic organization of the central auditory system and aims to desynchronize the abnormal tinnitus-related synchronized neural activity by periodically delivering sequences of randomly ordered sinusoidal tones with frequencies adapted to the tinnitus frequency (Tass et al., 2012a; Tass and Popovych, 2012). A randomized, single-blind, placebo-controlled 12-week proof of concept study in 63 patients with chronic subjective tonal tinnitus receiving aCR stimulation for 4–6 hours per day revealed significant therapeutic and electrophysiological effects compared to baseline (Tass et al., 2012a).

By design, effective CR stimulation requires phase resetting stimuli to be delivered to different subpopulations of a synchronized population of neurons (Tass, 2003). Accordingly, careful selection of the stimulation amplitude is key to the effects of CR. For instance, in parkinsonian nonhuman primates treated with CR deep brain stimulation (DBS) delivered to the subthalamic nucleus, it was shown that both acute effects (during five consecutive days with 2 hours of stimulation per day) and long-lasting after-effects (following cessation of stimulation) were weak when using strong stimulation amplitudes as are employed in conventional DBS. However, the effects became pronounced and lasted for a month when the stimulation amplitudes were one-third of those used in conventional DBS (Tass et al., 2012b). For a given spatial stimulation profile, the stimulation amplitude determines the amount of spatial overlap of the separately stimulated neuronal subpopulations (Tass, 2003; Manos et al., 2018). To that end, the present study aims to elucidate the optimal spatial stimulation profiles for aCR tones in order to achieve higher levels of desynchronization in the tinnitus focus of the auditory cortex.

For acoustic stimulation of the inner ear, auditory filters provide an equivalent concept to spatial stimulation profiles for electrical stimulation of neuronal tissue (Fletcher, 1940). Different auditory frequencies cause mechanical resonances at different points along the basilar membrane in the cochlea of the inner ear. Its spatially dependent mechanical sensitivity gives rise to a tonotopic organization of the basilar membrane to audio frequencies which can be represented as an array of “auditory filters”, i.e., overlapping band-pass filters (Fletcher, 1940), which continue to be represented tonotopically throughout the ascending auditory pathway to the level of the cortex. A second tone administered within the frequency range of an auditory filter causes auditory masking, i.e., it affects the perception of a first tone (Fletcher, 1940).

In psychoacoustics, the bandwidth of an auditory filter in human hearing is approximated by the corresponding equivalent rectangular bandwidth (ERB). An ERB describes a rectangular band-pass filter that passes the same amount of energy as its corresponding auditory filter (Moore and Glasberg, 1983; Glasberg and Moore, 1990). In this study, we use a mathematic model for tinnitus frequency and hearing impairment-induced increase of ERB width (Tass et al., 2019). This provides us with the possible range of spatial stimulation profiles achievable by regular aCR stimulation (Tass et al., 2012a). Second, given the validity and limitations of the mathematical ERB model (Tass et al., 2019), we here elucidate which spacing arrangements of CR tone ERBs may be favorable for tinnitus suppression. In particular, we focus on whether CR tones should be densely packed with corresponding ERBs overlapping or more widely spaced with gaps in between their ERBs. The ERB width depends on the ERB’s center frequency as well as the hearing impairment at that frequency (Moore and Glasberg, 1983; Glasberg and Moore, 1990; Tass et al., 2019). Hence, ERB widths of the original four CR tones (Tass et al., 2012a), as well as their mutual spatial arrangement, may vary depending on the tinnitus frequency and the hearing impairment.

On the whole, the findings presented herein are a re-analysis of the data acquired from 18 subjects with tinnitus from (Adamchic et al., 2017) who were treated with aCR stimulation. The method of re-analysis is based on the mathematical model of the ERB-scale, with dependencies on tinnitus frequency and hearing loss, described in (Tass et al., 2019). To detect favorable ERB spacing arrangements, we define in this manuscript a quantity called the “gap index” which quantifies the spacing between adjacent auditory filters. As will be described in the manuscript, the gap index serves as a comprehensive index of tone spacing, regardless of whether the ERBs of the CR tones have gaps or overlaps between them. We then assess the relationship of the gap index with the corresponding degree of clinical tinnitus suppression in a study of patients with chronic subjective tinnitus stimulated with two different variants of aCR stimulation which differ with respect to CR tone selection and, importantly, tone spacing (Adamchic et al., 2017). Regular aCR makes use of four tones with fixed spacing that is consistent across cycles. On the contrary, in noisy aCR, the tones are randomized prior to each cycle, resulting in a distinct spacing profile with each cycle (Tass et al., 2012a).

## Methods

### Participants

This study was approved by the Ethics Committee of Cologne University’s Faculty of Medicine. Written informed consent was obtained from all subjects according to the Declaration of Helsinki and Good Clinical Practice. Participants were 18 individuals with subjective bilateral chronic tonal tinnitus (15 males and 3 females). Individuals with pulsatile, buzzing, roaring, or hissing tinnitus and subjects with a history of auditory hallucinations, Ménière’s disease, middle ear disorders, and diagnosed neurological or mental disorders, as well as individuals taking CNS-acting medication or using hearing aids, were excluded. The mean age was 45.89 (±12.97 standard deviation, SD) years, and the mean tinnitus duration was 9.83 (±7.08) years. Otoscopic examination was performed in all participants. Additional details can be found in (Adamchic et al., 2017).

### Audiometric Testing

Extended high-frequency air conduction audiometry was performed in all subjects, ranging from 125 Hz to 16 kHz, with thresholds measured in dB HL. While estimates of the frequency distribution of the tinnitus pitch match (*f*_*T*_) vary across studies (Reed, 1960; Meikle and Taylor-Walsh, 1984; Savastano, 2004), the prevalence of *f*_*T*_ above 4,000 Hz in these studies is consistently sizeable. Thus, in order to comprehensively estimate hearing in the ranges possibly affected by the tinnitus, a five-frequency pure tone average (5-PTA) was calculated for each subject as the average of thresholds at 500, 1,000, 2000, 4,000, and 8,000 Hz.

The tinnitus pitch was determined by means of a pure tone matching procedure (from 0.5 to 13 kHz) (Adamchic et al., 2017). Starting from either well above or well below the subject’s tinnitus frequency, subjects had to adjust the frequency of a pure tone to the perceived pitch of their tinnitus. The tinnitus pitch matching procedure required patients to confirm the best matching pitch twice with a maximum modulus of the difference between two matched tones <100 Hz. Tinnitus pitches obtained ranged from 675 to 9,800 Hz. Audiometric data, tinnitus pitch, and tinnitus duration can be found in Supplementary Table S1.

### Stimulation Protocols and Symptom Scoring

**FIGURE 1:**
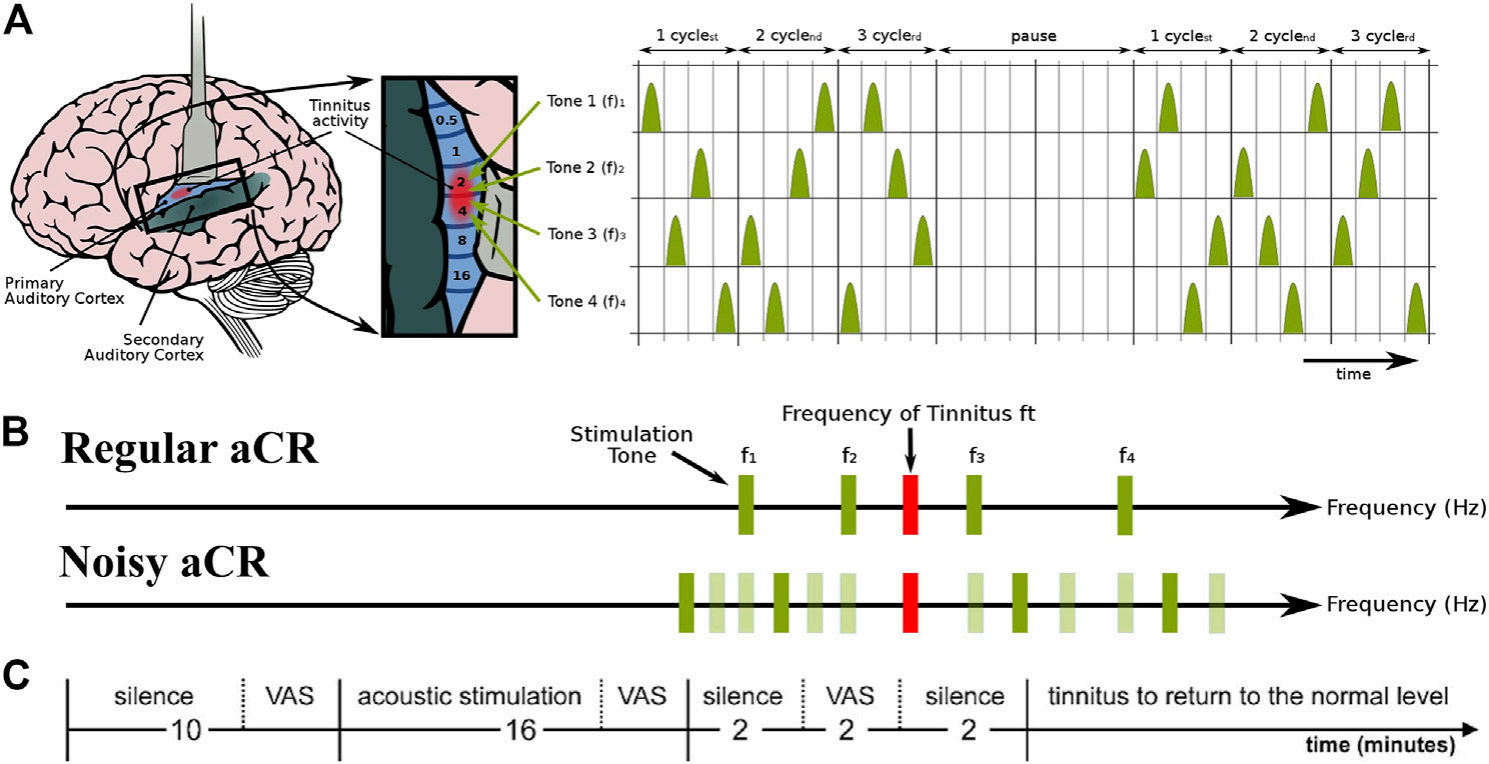
Schematic of the experimental paradigm and the two types of acoustic CR stimulation. **(A)** aCR stimulation pattern. For aCR, we employ the tonotopic organization of the primary auditory cortex (**left panel**, brain adapted from (Chittka and Brockmann, 2005) with kind permission of the authors) and deliver brief sinusoidal tones of different frequencies (pitch) f_1_, … , f_4_ equidistantly in time at a cycle repetition rate of 1.5 Hz (Tass et al., 2012a). Three CR cycles, each comprising a randomized sequence of four tones **(right panel)**, were followed by two silent cycles without stimuli (“pause”). The 3 cycles stim ON-2 cycles stim OFF pattern was repeated periodically (Tass, 2003; Tass and Majtanik, 2006; Lysyansky et al., 2011). Right panel from (Tass et al., 2012a) with kind permission by the authors. Copyright by Forschungszentrum Jülich GmbH. **(B)** Schematic illustration of the stimulus tones and repetition rates of regular aCR and noisy aCR. Panel partly redrawn from (Tass et al., 2012a) with kind permission by the authors. Copyright by Forschungszentrum Jülich GmbH. **(C)** Experimental session. During the first 10 min of silence, the baseline VAS-L and VAS-A scores were obtained. Thereafter, one of the two stimulation paradigms, i.e., regular aCR or noisy aCR, was performed for 16 min. VAS-L and VAS-A were obtained during stimulation and at the end of this stimulation period. After each session, participants received a pause during which tinnitus returned to the normal level. Thereafter the next session was started. Panel partly redrawn from (Adamchic et al., 2017) with kind permission by the authors. Copyright by Forschungszentrum Jülich GmbH.

Figure 1A illustrates the stimulation patterns for aCR. The aCR stimulation comprises the four tones whose frequencies are specified as fixed percentages relative to $f_{T\;}$ . There are two forms of aCR, referred to as “regular” and “noisy.” In *regular aCR*, two tones are placed below $f_{T\;}$ and two are placed above $f_{T\;}\;$ ( $f_{1}=0.76\cdot f_{T\;}$ , $\;f_{2}=0.9\cdot f_{T\;}$ , $f_{3}=1.1\cdot f_{T\;}$ , $f_{4}=1.4\cdot f_{T}$ ) (Figure 1B). For the *noisy aCR stimulation*, prior to each cycle, four frequencies $f_{1\;},\ldots .,\;f_{4}$ are randomly chosen out of a set of frequencies $c_{1\;}$ to $c_{12\;}$ with equal probability (Figure 1B) in the following way: $f_{1\;}$ is chosen from the set $S_{1}=\left\{c_{1},c_{2},c_{3}\right\},\;f_{2\;}$ is chosen from the set $S_{2}=\left\{c_{4},c_{5},c_{6}\right\}$ , $f_{3\;}$ is chosen from the set $S_{3}=\left\{c_{7},c_{8},c_{9}\right\}$ , and $f_{4\;}$ is chosen from the set $S_{4}=\left\{c_{10},c_{11},c_{12}\right\}$ , where $c_{j}=d_{j}f_{T}$ and $d_{1}=0.69,\;\;d_{2}=0.728,\;\;d_{3}=0.766,\;\;d_{4}=0.810,\;\;d_{5}=0.855,\;\;d_{6}=0.900,\;\;d_{7}=1.100,\;d_{8}=1.182,\;d_{9}=1.265,\;d_{10}=1.400,\;d_{11}=\;1.505,\;d_{12}=1.610$ . The duration of the acoustic tones is 150 ms for both protocols.

The frequency span for regular aCR stimulation reads $\;f_{4}-f_{1}=0.63\cdot f_{T}$ . For noisy aCR stimulation, the frequency span ranges from $\;c_{12}-c_{1}=0.920\cdot f_{T}$ to $\;c_{10}-c_{3}=0.634\cdot f_{T}$ with an average (over stimulation cycles) of $\bar{S_{4}}-\bar{S_{1}}=0.777\cdot f_{T}$ . Thus, the average frequency span of noisy aCR stimulation is approximately 23% greater than that of aCR stimulation, which means an even higher total span for the ERBs centered at the stimulation tone frequencies. In the case of aCR stimulation, the distances between adjacent tones are $\;f_{2}-f_{1}=0.134\cdot f_{T}$ , $\;f_{3}-f_{2}=0.2\cdot f_{T}$ , $\;f_{4}-f_{3}=0.3\cdot f_{T}$ . For the noisy aCR stimulation, the average distance between adjacent sets of tones is given by $\;\bar{S_{2}}-\bar{S_{1}}=0.127\cdot f_{T}$ , $\;\bar{S_{3}}-\bar{S_{2}}=0.327\cdot f_{T}$ , $\;\bar{S_{4}}-\bar{S_{3}}=0.323\cdot f_{T}\;$ , where $\;\bar{S_{1}},..,\bar{S_{4}}$ are the mean frequencies of the corresponding sets defined above. Thus, the distance between the inner sets of tones of the noisy aCR stimulation is on average 64% greater than the distance between the inner CR tones $f_{2}$ and $f_{3}$ . Accordingly, aCR stimulation is more closely spaced around the tinnitus frequency $f_{T}$ than is noisy aCR. More details about the frequency spacing for both protocols can be found in the article (Adamchic et al., 2017). For both stimulation protocols in this study, stimulation tones were equally loud. The loudness of the stimulation tones was set using the following procedure: First the intensity of the stimulation tone with the lowest frequency was set at threshold +20 dB. Loudness of the other stimulation tones was set so that they were perceived by the participant as equally loud as the first tone. Participants who were not able to hear all stimulation tones were excluded from the study.

The first part of each experimental session consisted of a 10-min period of silence (Figure 1C). The participants sat still and listened to their tinnitus. At the end of this baseline period, the participants were asked to indicate the mean tinnitus loudness and annoyance during the baseline resting period on a 100 mm long visual analogue scale (VAS) verbally anchored at the endpoints (Adamchic et al., 2012). VAS for loudness (VAS-L; tinnitus is not audible = 0, tinnitus is extremely loud = 100) and annoyance (VAS-A; tinnitus is not annoying = 0, tinnitus is extremely annoying = 100) were obtained. After the rating, CR stimulation (Figure 1B) was presented for exactly 16 min. At the end of this stimulation period, the participants were asked to indicate the mean tinnitus loudness and annoyance during the stimulation period on the VAS-L and VAS-A. The stimulus presentation was followed by a 2-min-long resting period with eyes closed, followed by a final VAS-L and VAS-A rating between 2 and 4 minutes after the stimulation. The order of the experimental sessions—whether regular aCR first or noisy aCR first—was pseudorandomly counterbalanced across subjects. The subjects thus underwent both types of aCR. The sequence of events in a typical experimental session is also illustrated in Figure 1C. Since the VAS-A and -L scores were measured both for the right and left ears, we used their arithmetic mean values normalized to their baseline values (Adamchic et al., 2017). Since the values were normalized to their baseline, the final score used in the analysis is a proportion (with 1.0 indicating a score unchanged from baseline). From this point on, all reference to VAS-A and VAS-L refers to the normalized score unless indicated otherwise. All subjects’ normalized VAS-L and VAS-A scores during and after therapy for regular and noisy aCR can be found in Supplementary Table S2.

### ERB Concept for Acoustic Stimulation

In the present study, we used a frequency scale based on the equivalent rectangular bandwidth (ERB) of the auditory filter, as determined from masking experiments using notched noise or spectrally rippled noise with human listeners (Moore and Glasberg, 1983; Glasberg and Moore, 1990). The average value of the ERB for young listeners with normal audiometric thresholds measured at moderate sound levels is denoted as ERB_N_. Its value in Hz is given by

$$\;ERB_{N}=24.7\cdot \left(0.00437\cdot f+1\right),$$ (1)

where f is the center frequency in Hz (Glasberg and Moore, 1990). This equation provides a good prediction of ERB values estimated psychoacoustically using masking experiments for center frequencies spanning almost the entire range of human hearing from about 50 to 15,000 Hz (Zhou, 1995). The edges of the ERB-wide frequency intervals were estimated at low sensation levels using data presented by Moore and others, where the value of the ERB was provided for center frequencies 2,000, 4,000, and 6,000 Hz and audiometric thresholds from 0 to 80 dB HL (Moore et al., 1999). From these data, the following equation was generated for hearing loss h in the range of 0–50 dB HL (Tass et al., 2019):

$$ERB\left(h\right)=ERB_{N}\cdot \left(1+h/50\;dB\;HL\right),$$ (2)

The values of frequencies at the upper edges $a_{T}$ and lower edges $b_{T}$ of the ERB-wide band centered around $f_{T\;}$ are defined by

$$a_{T}=f_{T}-0.5\cdot ERB\left(h\left(f_{T}\right)\right),$$ (3)

$$b_{T}=f_{T}+0.5\cdot ERB\left(h\left(f_{T}\right)\right),$$ (4)

Analogously, the frequencies at the upper and lower edges of the ERB band centered at the frequency of CR tone j, $a_{j}$ , and $b_{j}$ , read

$$a_{j}=f_{j}-0.5\cdot ERB\left(h\left(f_{j}\right)\right),$$ (5)

$$b_{j}=f_{j}+0.5\cdot ERB\left(h\left(f_{j}\right)\right),$$ (6)

**FIGURE 2:**
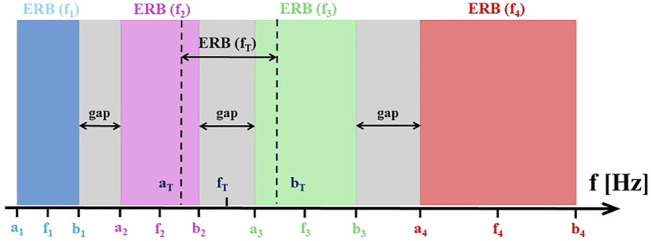
The alignment of CR tones, tinnitus tone $f_{T\;}$ and corresponding ERBs in the case when the ERBs of neighboring CR tones are non-overlapping. The symbols used to designate the band edges and center frequencies of the ERBs around the center frequencies of each of the four original CR tones and $f_{T\;}$ . The gaps between the ERBs centered at the CR tones are illustrated by gray stripes.

Figure 2 illustrates the alignment of CR tones $f_{1},\ldots f_{4}$ , tinnitus tone $f_{T\;}$ together with the corresponding ERBs for the case when the ERBs of neighboring CR tones are not overlapping.

### Gap Index for Psychophysical ERBs

#### Gaps and Overlaps for Regular aCR Stimulation

For subjects with normal hearing, neighboring ERBs of CR tones do not overlap for tinnitus frequencies $f_{T\;}$ greater than 560 Hz (Tass et al., 2019). With increasing hearing impairment, the width of the gaps between the CR tone ERBs may decrease or even vanish (Tass et al., 2019). To quantify the spacing of the CR tones, we introduce different quantities measuring gaps and overlaps. To this end, we introduce the *relative gap* between $ERB\left(f_{j}\right)$ and $ERB\left(f_{k}\right)$ , where $f_{j}<f_{k}$ , by

$$G\left(f_{j},f_{k}\right)=\;\;\frac{G_{abs}\left(f_{j},f_{k}\right)}{ERB\left(f_{k}\right)}=\frac{a_{k}-b_{j}}{ERB\left(f_{k}\right)}\;,$$ (7)

where $G_{abs}\left(f_{j},f_{k}\right)$ denotes the absolute gap between $ERB\left(f_{j}\right)$ and $ERB\left(f_{k}\right)$ , where $f_{j}<f_{k}$ . $G\left(f_{j},f_{T}\right)>0$ means that $ERB\left(f_{j}\right)$ and $ERB\left(f_{k}\right)$ do not overlap and are separated by a gap, whereas $G\left(f_{j},f_{k}\right)<0$ , if $ERB\left(f_{j}\right)$ and $ERB\left(f_{k}\right)$ overlap. $G\left(f_{j},f_{k}\right)=0$ if both ERBs touch each other, so that the edges of $ERB\left(f_{j}\right)$ and $ERB\left(f_{k}\right)$ coincide.

For regular aCR stimulation, the *mean relative gap* between all pairs of neighboring ERBs, $ERB\left(f_{j}\right)$ and $ERB\left(f_{j+1}\right)$ (with j=1,2,3 ), reads

$$\bar{G}=\overset{3}{\underset{j=1}{\sum }}G\left(f_{j},f_{j+1}\right),$$ (8)

For regular aCR stimulation, the entire frequency range spanned by the ERBs centered around four CR tones is given by the *ERB span* $E=\left[a_{1},\;b_{4}\right]$ (Figure 2). As an alternative means to assess the spacing of the CR tone ERBs, we introduce the *gap index* g by

$$g=\;\left[\overset{3}{\underset{j=1}{\sum }}\overset{3}{\underset{j=1}{\sum }}\left[G_{abs}\left(f_{j},f_{j+1}\right)\right]{}_{+}\right]/\left(b_{4}-a_{1}\right),$$ (9)

where ${\left[\ldots \right]}_{+}$ is defined by

$${\left[x\right]}_{+}=\{\begin{array}{c} \;x\;:x>0\\ \;0\;:x\leq 0 \end{array}\;.$$

Thus, the gap index provides us with the percentage of E covered by the gray stripes as shown in Figure 2.

As shown in Supplementary Figure 1A, the relative gap $G\left(f_{j},f_{j+1}\right)$ is highly correlated with the gap index (with the *r* ranging from 0.92 to 0.99, depending on which tone pair is assessed), regardless of whether the relative gap is negative, zero, or positive. Further, as shown in Supplementary Figure 2A, the mean relative gap $\bar{G}$ between all pairs of neighboring ERBs is also highly correlated with the gap index *g* (*r* = 0.99). This motivates the use of the gap index as a summary index of the spacing of the ERBs of all four CR tones, rather than the behavior of individual pairwise ERBs.

In addition, we determine the number of gaps (i.e., gray stripes in Figure 2) between the four ERBs belonging to the regular aCR tones. To this, in analogy to Eq. 7 we determine whether there is a gap between $ERB\left(f_{j}\right)$ and $ERB\left(f_{k}\right)$ , where $f_{j}<f_{k}$ , by

$$H\left(j,k\right)=\{\begin{array}{c} 1\;:a_{k}-b_{j}>0\\ 0\;:else \end{array},$$ (10)

H(j,k)=1 if there is a gap between $ERB\left(f_{j}\right)$ and $ERB\left(f_{k}\right)$ and 0 else. To count the number of gaps between neighboring ERBs belonging to the regular aCR tones, we introduce the *gap count* c is then defined by

$$c=\overset{3}{\underset{j=1}{\sum }}H\left(j,j+1\right),$$ (11)

$c\left(f_{T},h\right)$ can attain integer values between 0 and 3. Supplementary Figure 3A demonstrates the relationship between the gap count *c* and the gap index *g* (*r* = 0.94).

Gap index and gap count quantify the overall spacing of the entire ERB arrangement of the CR therapy tones. In addition, as an alternative means to describe the spatial CR tone arrangement, we use the tinnitus $ERB\left(f_{T}\right)$ as reference. To assess to which extent the tinnitus $ERB\left(f_{T}\right)$ is covered by $ERB\left(f_{j}\right)$ of the CR therapy tones j=1,2,3,4 , we determine the *relative overlap between* the tinnitus $ERB\left(f_{T}\right)$ and the CR tone $ERB\left(f_{j}\right)$

$$O\left(f_{j},f_{T}\right)=\;\{\begin{array}{c} \left(b_{j}-a_{T}\right)/ERB\left(f_{T}\right)\;:\;f_{j}<f_{T}\\ \left(b_{T}-a_{j}\right)/ERB\left(f_{T}\right)\;:f_{j}>f_{T} \end{array}\;,$$ (12)

$O\left(f_{j},f_{T}\right)>0$ means that $ERB\left(f_{T}\right)$ and $ERB\left(f_{j}\right)$ overlap, whereas $O\left(f_{j},f_{T}\right)<0$ corresponds to $ERB\left(f_{T}\right)$ and $ERB\left(f_{j}\right)$ being separated by a gap. $O\left(f_{j},f_{T}\right)=0$ if $ERB\left(f_{T}\right)$ and $ERB\left(f_{j}\right)$ touch each other with coincident ERB edges.

With this the *mean relative overlap* between the tinnitus $ERB\left(f_{T}\right)$ and all other CR tone ERBs, $ERB\left(f_{1}\right),\ldots ,ERB\left(f_{4}\right)$ , reads

$$\bar{O}\left(f_{T}\right)=\overset{4}{\underset{j=1}{\sum }}O\left(f_{j},f_{T}\right),$$ (13)

As shown in Supplementary Figure 4A, the relative overlap and the gap index are highly correlated (*r* ranges from −0.99 to −0.95, depending on which tone pair is assessed). As shown in Supplementary Figure 5A, the mean relative overlap is also highly correlated with the gap index (*r* = −0.98). Thus, the overlaps of the CR tone ERBs with the tinnitus ERB are also captured by the gap index.

The advantage of using the gap index, a single, integral spacing measure for all CR tones (rather than, for example, multiple such metrics for single ERB pairs) is that the gap index reflects the spacing of CR tone ERBs in their entirety and enables a comprehensive correlation to different clinical outcome measures. It also captures the relationship of CR tone ERBs to the tinnitus ERB.

#### Gaps and Overlaps for Noisy aCR Stimulation

To determine the mean gap between all pairs of CR tone frequencies $f_{j\;}$ and $f_{k}$ , we first determine the *relative gap* defined by Eq. 7 according to

$$G_{N}\left(c_{m},c_{n}\right)=\frac{a_{n}-b_{m}}{ERB\left(c_{n}\right)},$$ (14)

for $c_{m}\in S_{j}$ and $c_{n}\in S_{k}$ , and $a_{n}$ and $b_{n}$ denote the lower and upper edge of $ERB\left(c_{n}\right)$ (see Figure 2).

With this, the *mean relative gap* between all pairs of neighboring CR frequencies $f_{j\;}$ and $f_{j+1}$ (j=1,2,3) for noisy aCR stimulation reads

$${\bar{G}}_{N}\left(f_{j},f_{j+1}\right)=\underset{c_{m}\in S_{j},\;c_{n}\in S_{k}}{\sum }G\left(c_{m},c_{n}\right),$$ (15)

For noisy aCR stimulation, the ERB span depends on the lowest and highest CR tone of the respective noisy aCR cycle (see *Stimulation Protocols and Symptom Scoring*). To determine the gap index for noisy aCR stimulation, for each realization of noisy aCR cycles we consider the percentage ERB span consisting of gaps in between the CR tone ERBs and average over all (equally frequent) possible realizations, where the set of realizations is given by

$$\begin{array}{l} ℛ={\left\{\left(f_{j},f_{k},f_{l},f_{m}\right)\right\}}_{j=1,2,3;k=4,5,6;l=7,8,9;m=10,11,12}\\ =:\;{\left\{\left(j,k,l,m\right)\right\}}_{j=1,2,3;k=4,5,6;l=7,8,9;m=10,11,12} \end{array}$$ (16)

(see Stimulation Protocols and Symptom Scoring). To this end, we use Eq. 7 for the absolute gap $G_{abs}\left(f_{j},f_{k}\right)\;$ between $ERB\left(f_{j}\right)$ and $ERB\left(f_{k}\right)$ and calculate the gap index $g\left(f_{T},h\right)$ for each single realization (j,k,l,m) . Denoting the gap index for realization (j,k,l,m)  by $g\left(f_{T},h,j,k,l,m\right)$ , the *gap index for noisy aCR* reads

$$\bar{g}\left(f_{T},h\right)=\frac{1}{81}\underset{j=1,2,3;k=4,5,6;l=7,8,9;m=10,11,12}{\sum }g\left(f_{T},h,j,k,l,m\right),$$ (17)

The relationship of relative gap to gap index for noisy aCR is shown in Supplementary Figure 1B (*r* ranges from 0.93 to 0.98 depending on which tone pair is assessed) and mean relative gap to gap index for noisy aCR in Supplementary Figure 2B (*r* = 0.999).

For noisy aCR stimulation, we additionally determine the *gap count*

$$\bar{c}\left(f_{T},h\right)=\frac{1}{81}\underset{j=1,2,3;k=4,5,6;l=7,8,9;m=10,11,12}{\sum }c\left(f_{T},h,j,k,l,m\right),$$ (18)

To this end, we replace the term for the gap, $a_{k}-b_{j}$ , by the corresponding Heaviside function H(j,k) (Eq. 10). Supplementary Figure 3B demonstrates the relationship between the gap count $\bar{c}$ and the gap index $\bar{g}$ (*r* = 0.97).

In addition, we describe the spatial CR tone arrangement by using the tinnitus $ERB\left(f_{T}\right)$ as reference. To assess to which extent the tinnitus $ERB\left(f_{T}\right)$ is covered by $ERB\left(c_{m}\right)$ , in analogy to Eq. 12, we determine the *relative overlap between* the tinnitus $ERB\left(f_{T}\right)$ and $ERB\left(c_{m}\right)$

$$O\left(c_{m},f_{T}\right)=\;\{\begin{array}{c} \left(b_{m}-a_{T}\right)/ERB\left(f_{T}\right)\;:\;c_{m}<f_{T}\\ \left(b_{T}-a_{m}\right)/ERB\left(f_{T}\right)\;:c_{m}>f_{T} \end{array},$$ (19)

For noisy aCR stimulation, the *mean relative overlap* between all three realizations of a CR frequency $f_{j\;}$ and the tinnitus $ERB\left(f_{T}\right)$ reads

$$O_{N}\left(f_{j},f_{T}\right)=\underset{c_{m}\in S_{j}}{\sum }O\left(c_{m},f_{T}\right),$$ (20)

With this the *mean relative overlap* between the tinnitus $ERB\left(f_{T}\right)$ and all other CR tone ERBs, $ERB\left(f_{1}\right),\ldots ,ERB\left(f_{4}\right)$ , reads

$${\bar{O}}_{N}\left(f_{T}\right)=\overset{4}{\underset{j=1}{\sum }}O_{N}\left(f_{j},f_{T}\right),$$ (21)

The relative overlap vs. gap index for noisy aCR is shown in Supplementary Figure 4B (*r* ranges from −0.97 to −0.95 depending on which tone pair is assessed). The mean relative overlap vs. gap index for noisy aCR is shown in Supplementary Figure 5B (*r* = −0.97). As is the case with regular CR, the gap index once again represents a single measure that captures multiple relationships among the noisy aCR tone ERBs and between the noisy aCR tone ERBs to the tinnitus ERB.

### Spacing of CR Tones

According to the American Speech-Language-Hearing Association (ASHA), the hearing thresholds relevant to our study can be classified with the following descriptors: normal hearing (0–15 dB), slight hearing loss (16–25 dB), mild hearing loss (26–40 dB), and moderate hearing loss (41–55 dB) (Goodman, 1965; Clark, 1981). Due to deterioration in estimates of the ERB width beyond hearing thresholds of 50 dB, we do not plot severe-to-profound hearing loss here. Nine of the eighteen subjects had at least one threshold above 50 dB in at least one ear at the frequencies included in the 5-PTA.

**FIGURE 3:**
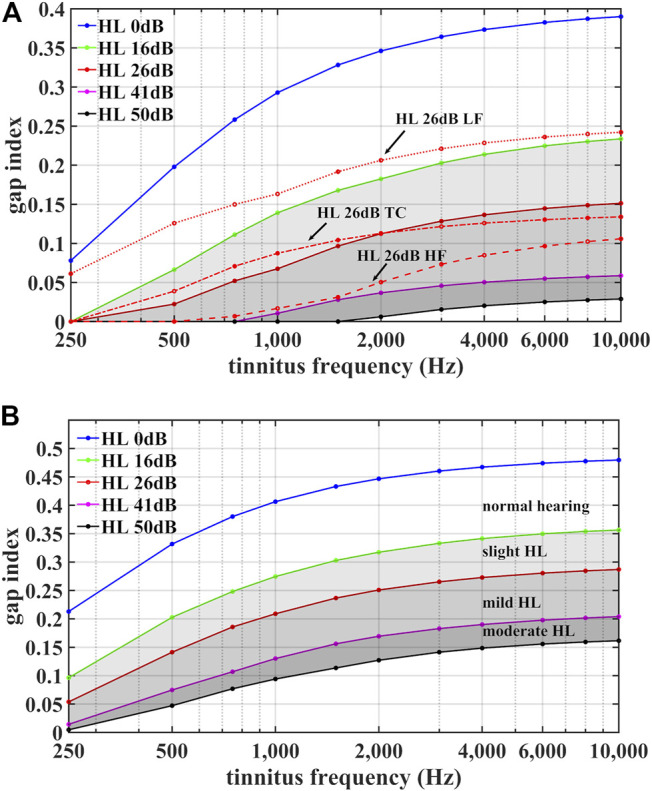
Illustration of the range of possible gap index values for regular aCR stimulation **(A)** and for noisy aCR stimulation **(B)**. The gap indices $g\left(f_{T},h\right)$ from Eq. 9 **(A)** and $\bar{g}\left(f_{T},h\right)$ from Eq. 17 **(B)** are plotted against tinnitus frequency $f_{T\;}$ for homogenous hearing loss of 0 dB (“HL 0 dB”), 16 dB (“HL 16 dB”), 26 dB (“HL 26 dB”), 41 dB (“HL 41 dB”), 50 dB (“HL 50 dB”) at all corresponding CR tones, delineating ranges of maximum gap index for normal hearing as well as slight, mild, and moderate hearing loss. Additionally, for regular aCR in part **(A)**, we plot curves “HL 26 dB LF,” “HL 26 dB HF,” and “HL 26 dB TC,” to demonstrate how alternatively shaped audiograms with hearing loss in the lower-frequency, higher-frequency, and tinnitus-centered regions, respectively, affect the gap index. Despite all audiograms having average hearing loss of 26 dB, the shape of the audiogram is of critical importance in determining the gap index.

To illustrate how the range of possible gap index values for aCR stimulation depends on tinnitus frequency $f_{T\;}$ and hearing loss, we plot the gap index against tinnitus frequency $f_{T\;}$ , assuming homogenous hearing loss, where 0, 16, 26, and 41 dB hearing loss applies to all corresponding CR frequencies, respectively (“HL 0dB”, …, “HL 41dB” in Figure 3A). In these cases, $h\left(f_{1}\right),\ldots ,h\left(f_{4}\right)=\;$ 0, 16, 26, and 41 dB, respectively. This is to illustrate maximum ranges of the gap index belonging to normal hearing as well as slight, mild, and moderate homogenous hearing loss. Analogously, we plot the same curves for noisy aCR stimulation (Figure 3B). The maximum range of possible gap index values increases with increasing tinnitus frequency and begins to broaden even for milder degrees of homogenous hearing loss. The tinnitus frequency-dependent increase is more pronounced for aCR stimulation than for noisy aCR stimulation. In general, the values of the gap index for aCR stimulation are smaller than for noisy aCR stimulation.

However, the gap index not only depends on the average hearing loss in the vicinity of the tinnitus frequency $f_{T}$ (i.e., the frequency range between $f_{1},\;$ the lowest CR tone, and $f_{4},\;$ the highest CR tone) but may strongly vary depending on the actual shape of the audiogram in that frequency range. For illustration, for aCR stimulation we consider different types of non-homogenous hearing impairment. For this, we introduce the mean hearing impairment belonging to all four CR tones by

$$\bar{h}\left(f_{T}\right)=\frac{1}{4}\overset{4}{\underset{j=1}{\sum }}h\left(f_{j}\right)\;.$$

We consider different shapes of (model) audiogram with the same mean $\bar{h}\left(f_{T}\right)$ as for the homogenous hearing impairment at 26 dB shown in Figure 3A:

On average 26 dB hearing loss:

For $\bar{h}\left(f_{T}\right)=26$ dB we consider three different cases with non-homogenous (model) hearing impairment (Figure 3A):

1) Lower-frequency hearing loss (“HL 26 dB LF”): $h\left(f_{1}\right)=h\left(f_{2}\right)=50$ dB, $h\left(f_{3}\right)=4$ dB, $h\left(f_{4}\right)=$ 0 dB.
2) Higher-frequency hearing loss (“HL 26 dB HF”): $h\left(f_{4}\right)=h\left(f_{3}\right)=50$ dB, $h\left(f_{2}\right)=4$ dB, $h\left(f_{1}\right)=$ 0 dB.
3) Tinnitus-centered hearing loss (“HL 26 dB TC”): $h\left(f_{3}\right)=50$ dB, $h\left(f_{2}\right)=14$ dB, $h\left(f_{1}\right)=h\left(f_{4}\right)=$ 0 dB.

As shown in Figure 3A, the lower-frequency (model) hearing impairment has the greatest gap values, even exceeding the HL 16 dB curve for homogenous hearing impairment. Conversely, for frequencies between 800 Hz and 1,500 Hz the gap index of the higher-frequency (model) hearing impairment approaches gap index values of the HL 41 dB curve for homogenous hearing impairment. Similar audiogram-shape dependent variations of the gap index were also observed for $\bar{h}\left(f_{T}\right)=16$ dB and $\bar{h}\left(f_{T}\right)=41$ dB, as described in the Supplementary Methods. In summary, the gap index does not simply depend on the average hearing impairment around $f_{T}$ . The gap index rather strongly depends on the actual shape of the audiogram in the vicinity of the tinnitus frequency $f_{T}.$

### Calculation of Gap Index

To compute the distances between adjacent ERBs and the overall gap index (Eq. 9, above) for the aCR tones used in the clinical study, we used the following procedure:

1) The audiogram of every tinnitus patient was linearly interpolated on a logarithmic frequency axis.
2) The frequencies of the tones used for regular CR stimulation and for noisy aCR stimulation were computed for every tinnitus patient, and the hearing thresholds at the frequencies of the stimulation tones were interpolated from the audiometric thresholds.
3) The edges of the ERB-wide frequency intervals centered at the frequencies of CR tones were estimated using Eqs. 5, 6.
4) The gaps between the ERBs centered at the neighboring CR tones were calculated using Eq. 7 for every tinnitus patient.
5) The values of the gap index were calculated using Eq. 9 for every tinnitus patient in the case of regular aCR stimulation and Eq. 17 for noisy aCR stimulation.
a) Notably, if the tinnitus frequency was the same in both ears, the gap index for both ears was calculated with the mean values of the linearly interpolated audiograms of both ears.
b) However, if the tinnitus frequency was different between the ears, the gap index was initially calculated for each ear separately (using the individual thresholds for each ear). Then, the two indices for the ears were averaged for the final gap index.

### Statistical Analysis

All statistical analyses were performed using IBM SPSS Statistics (2020).

#### Primary Analysis

VAS-L score during stimulation was chosen as the primary outcome variable. This was motivated by several factors. Firstly, the most pronounced clinical effect seen in Adamchic et al., 2017 was the acute effect on loudness and annoyance. Stimulation-induced reduction of VAS-L and VAS-A was quite similar (Adamchic et al., 2017). However, tinnitus loudness appears to be the more elementary measure as opposed to tinnitus annoyance, the latter possibly prone to complex psychological factors (Hiller and Goebel, 2007; Guimaraes et al., 2014), such as a high degree of self-attention and somatic attention (Newman et al., 1997). Additionally, the score during stimulation rather than after stimulation was chosen as the primary outcome variable. The goal of CR stimulation is to induce long-lasting effects. However, this requires stimulation of sufficient duration. Based on the 2012 proof-of-concept study (Tass et al., 2012a), we could not expect to induce full-scale effects after only 16 min. In addition, it is not known to which extent the required stimulus duration depends on factors like disease duration. Onset and time course of neuronal plastic changes may depend on several factors. From computational studies in simple neural networks, we know that acute after-effects reflect the CR stimulation-induced changes and reduction of synaptic weights (Khaledi-Nasab, 2021). However, we focus on the most pronounced acute effects that may be less dependent on several other factors intrinsic to patients. Thus, overall, the VAS-L score during stimulation may be a relatively “purer” metric of stimulus efficacy less dependent on patient-specific factors, more so than the VAS-A score during or after stimulation, or VAS-L score after stimulation.

The primary analysis thus included linear regression models to determine the effects of tinnitus duration, gap index, and 5-PTA on the VAS-L score during regular and noisy aCR. Bi-variate correlations amongst the predictor variables demonstrated multicollinearity of the gap index and 5-PTA. For aCR, the gap index and 5-PTA had an *r* = −0.659 for regular aCR. The gap index and 5-PTA had an *r* = −0.705 for noisy aCR. This indicates that higher 5-PTA is correlated with lower gap indices, as would be expected due to broadening of ERBs with increasing threshold. Because the 5-PTA is a function of the patient’s underlying hearing loss and cannot be modified when selecting CR parameters, we chose the gap index as the more preferred predictor variable in this study over 5-PTA. To that end, we calculated the residual variance of 5-PTA toward the response variable of gap index for both regular aCR and noisy aCR. Residual variance is the unexplained variance between 5-PTA and the gap index. This method enabled us to correct for multicollinearity between these predictor variables. Of note, patient age was not included in the regression models, as hearing loss has a known significant impact on cochlear tuning, while age may have a smaller impact (Lutman et al., 1991). The bivariate correlation between the two variables of age and 5-PTA demonstrated an *r* = 0.635. Thus, 5-PTA was preferentially selected over age to minimize further multicollinearity, considering the known smaller effect of age on cochlear tuning relative to the effect of hearing loss.

Gap index, tinnitus duration, and residual variance of 5-PTA (calculated from the relationship to the gap index of either regular or noisy aCR) were included in the models with the VAS-L during therapy as the primary outcome variable.

#### Secondary Analyses

Additionally, we chose to perform secondary analyses that were exploratory in nature and therefore had no formal hypotheses. The secondary analyses were performed with the outcome variables of VAS-L after stimulation, as well as VAS-A during and after stimulation. Furthermore, the mean relative overlap and mean relative gap were also assessed as part of the secondary analyses. As in the primary analysis, the 5-PTA was residualized in all secondary analyses to account for multicollinearity.

## Results

### Primary Analysis

**FIGURE 4:**
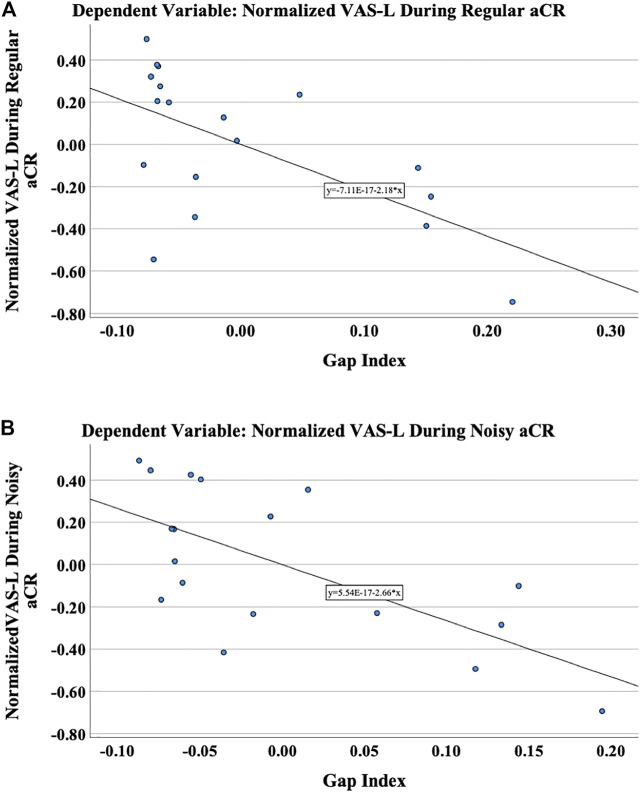
The primary outcome variable was chosen to be the normalized VAS-L during therapy. These are the partial regression plots demonstrating the relationship of the residuals of the normalized VAS-L to the residuals of the gap index after regressing on the residual variance of 5-PTA during **(A)** regular aCR and **(B)** noisy aCR. It should be noted that partial regression plots demonstrate the residuals of the represented variables, rather than the variables themselves.

As shown in Table 1, in the linear models for VAS-L during both regular and noisy aCR, the gap index was the only significant predictor variable. Partial regression plots for the primary analysis are shown in Figure 4. It should be noted that partial regression plots demonstrate the residuals of the represented variables, rather than the variables themselves. For this reason, the *y*-axis in each subfigure of Figure 4 represents the residual of VAS-L during CR, and the *x*-axis represents the residual of the gap index after removing the linear effects of the residual variance of 5-PTA. The slope of each plot is the same as the unstandardized beta coefficient (*B*) for the gap index for each multiple linear regression, the values of which can be found in Table 1.

**TABLE 1:** Multiple linear regression models using tinnitus duration, gap index, and residual variance of 5-PTA to gap index as predictor variables. The primary outcome variable chosen was VAS-L during therapy. The additional variables of VAS-L after therapy and VAS-A during and after therapy are also shown. Findings are shown for both regular aCR and noisy aCR. *B* (*SE*) represents unstandardized beta with standard error in parentheses.

VAS-L during therapy					
**Regular aCR (*n* =18, *R* ^2^ = 0.582, *F*(3,14) = 6.490, *p* =0.006^a^)**	***B* (*SE*)**	**β**	**Partial**	**Part**	***p*-value**
Constant	0.595 (0.162)				0.003^a^
Duration	0.002 (0.001)	0.350	0.415	0.295	0.110
Gap index	−2.175 (0.754)	−0.499	−0.611	−0.499	0.012^a^
Residual variance of 5-PTA	0.020 (0.014)	0.295	0.359	0.249	0.172
**Noisy aCR (*n* =18, *R* ^2^ = 0.551, *F*(3,14) = 5.730, *p* =0.009^a^)**	***B* (*SE*)**	**β**	**Partial**	**Part**	***p*-value**
Constant	1.097 (0.238)				<0.001^a^
Duration	0.001 (0.001)	0.216	0.268	0.187	0.315
Gap index	−2.657 (0.786)	−0.609	−0.670	−0.605	0.004^a^
Residual variance of 5-PTA	0.016 (0.014)	0.240	0.296	0.208	0.265
**VAS-L AFTER THERAPY**					
**Regular aCR (*n* =18, *R* ^2^ = 0.442, *F* (3,14) = 3.696, *p* =0.038^a^)**	***B* (*SE*)**	**β**	**Partial**	**Part**	***p*-value**
Constant	0.642 (0.129)				<0.001^a^
Duration	0.002 (0.001)	0.511	0.499	0.431	0.049^a^
Gap index	−1.020 (0.600)	−0.339	−0.413	−0.339	0.111
Residual variance of 5-PTA	0.004 (0.011)	0.085	0.095	0.071	0.726
**Noisy aCR (*n* =18, *R* ^2^ = 0.437, *F* (3,14) = 3.617, *p* =0.040^a^)**	***B* (*SE*)**	**β**	**Partial**	**Part**	***p*-value**
Constant	1.022 (0.251)				0.001^a^
Duration	0.001 (0.001)	0.277	0.304	0.240	0.252
Gap index	−2.125 (0.828)	−0.518	−0.566	−0.515	0.022^a^
Residual variance of 5-PTA	0.010 (0.015)	0.151	0.172	0.131	0.524
**VAS-A DURING THERAPY**					
**Regular aCR (*n* =18, *R* ^2^ = 0.674, *F*(3,14) = 9.633, *p* =0.001^a^)**	***B* (*SE*)**	**β**	**Partial**	**Part**	***p*-value**
Constant	0.647 (0.162)				0.001^a^
Duration	0.002 (0.001)	0.357	0.466	0.301	0.069
Gap index	−2.726 (0.750)	−0.555	−0.697	−0.555	0.003^a^
Residual variance of 5-PTA	0.025 (0.014)	0.319	0.426	0.269	0.100
**Noisy aCR (*n* =18, *R* ^2^ = 0.530, F(3,14) = 5.270, *p* =0.012^a^)**	***B* (*SE*)**	**β**	**Partial**	**Part**	***p*-value**
Constant	0.968 (0.244)				0.001^a^
Duration	0.001 (0.001)	0.276	0.328	0.238	0.215
Gap index	−2.424 (0.806)	−0.554	−0.626	−0.551	0.009^a^
Residual variance of 5-PTA	0.016 (0.015)	0.234	0.284	0.203	0.286
**VAS-A AFTER THERAPY**					
**Regular aCR (*n* =18, *R* ^2^ = 0.699, *F*(3,14) = 10.841, *p* <0.001^a^)**	***B* (*SE*)**	**β**	**Partial**	**Part**	***p*-value**
Constant	0.689 (0.140)				<0.001^a^
Duration	0.003 (0.001)	0.489	0.600	0.412	0.014^a^
Gap index	−2.566 (0.649)	−0.580	−0.726	−0.580	0.001^a^
Residual variance of 5-PTA	0.011 (0.012)	0.153	0.230	0.129	0.392
**Noisy aCR (*n* =18, *R* ^2^ = 0.449, *F*(3,14) = 3.804, *p* =0.035^a^)**	***B* (*SE*)**	**β**	**Partial**	**Part**	***p*-value**
Constant	1.090 (0.254)				<0.001^a^
Duration	0.001 (0.001)	0.260	0.289	0.224	0.278
Gap index	−2.360 (0.838)	−0.562	−0.601	−0.559	0.014^a^
Residual variance of 5-PTA	0.007 (0.015)	0.105	0.122	0.091	0.652

a Denotes statistically significant result for alpha level <0.05.

### Secondary Analyses

#### Gap Index

As shown in Table 1 and Supplementary Table 6, in the linear models for VAS-L after regular aCR, only the duration was a significant predictor variable. However, in noisy aCR, the gap index was once again significant. In looking at VAS-A during therapy, the gap index was significant for both regular and noisy aCR. In looking at the VAS-A after therapy, both the duration and gap index were significant for regular aCR, while only the gap index was significant for noisy aCR.

#### Mean Relative Gap

As shown in Supplementary Table 4 and Supplementary Table 6, the findings for the mean relative gap were very similar to those seen with the gap index. The mean relative gap was a significant predictor variable for all models except the VAS-L after regular aCR, in which only the duration was significant. For VAS-A after regular and noisy aCR, both the duration and mean relative gap were significant.

#### Mean Relative Overlap

As shown in Supplementary Table 5 and Supplementary Table 6, for VAS-L during regular aCR, only the residual variance of 5-PTA was significant. For VAS-L during noisy aCR, the mean relative overlap was significant. For VAS-L after regular aCR, the duration was trending toward significance, while in noisy aCR, the duration was again trending, with mean relative overlap as significant. For VAS-A during regular aCR, the mean relative overlap and residual variance of 5-PTA were significant. For VAS-A during noisy aCR, only the mean relative overlap was significant. For VAS-A after regular and noisy aCR, the duration and mean relative overlap were both significant.

#### Tone Spacing Relationships

Some additional findings from observing tone spacing relationships are described herein. Notably, only for regular aCR do the pairwise relative gaps increase with increasing tone frequency, as seen in Supplementary Figure 1 (i.e., in (A), G (1,2)< G(2,3)<G(3,4)  on average, while in (B), G (1,2)< G(3,4)<G(2,3) . Additionally, when looking at the relative overlap, as in Supplementary Figure 4, for regular aCR, even at large values of the gap index, there is overlap of the ERBs for tones 2 and 3 with the tinnitus ERB. In noisy aCR, however, the ERBs for tones 2 and 3 do not overlap with the tinnitus ERB.

## Discussion

### Overall Findings

Coordinated reset was developed computationally to lead to desynchronization of populations of neurons with abnormal degrees of coincident firing (Tass, 2003; Tass and Popovych, 2012). However, the optimal values of overlap of spatial stimulation profiles for aCR—leading to maximal desynchronization and clinical benefit—remain an unknown in clinical practice. Several lines of evidence suggest that stimulus amplitudes and the intimately associated parameter of spatial stimulation profiles can have major impacts on the degree of desynchronization achievable. Tass et al. demonstrated that CR-DBS with one-third the stimulus amplitude of conventional DBS leads to more pronounced clinical effects in parkinsonian monkeys (Tass et al., 2012b). Additional computational studies have demonstrated the presence of the “spacing principle,” whereby spaced CR stimulation at weaker intensities can effectively induce anti-kindling (Popovych et al., 2015).

This study aims to shed light on the impact of tone spacing on clinical efficacy of regular and noisy aCR, as measured by the changes to self-reports of tinnitus loudness and annoyance during and after stimulation. The existing tone paradigms were transformed into an ERB-scale, which allowed for an analysis of the impact of aCR spatial stimulation profiles on clinical outcome. The gap index—or fraction of the target spectral range uncovered by stimulation—was introduced as a measure of spacing between adjacent auditory filters. We carefully analyzed several possible metrics, as described in the Methods, and found the gap index to be a useful composite measure that captures multiple relationships, both among the therapeutic tones and also between the therapeutic tones and the tinnitus frequency.

To that end, several multiple linear regression models were developed, using tinnitus duration, the gap index, and residual variance of 5-PTA (after controlling for multicollinearity with the gap index) as predictor variables. The primary outcome variable was VAS-L during therapy, with additional secondary analyses performed on the response variables of VAS-L after therapy and VAS-A during and after stimulation with the two CR paradigms. The gap index was found to be the primary predictor variable for the primary outcome variable of VAS-L during regular aCR and during noisy aCR. This suggests that, as the gap index becomes smaller, i.e., the ERBs corresponding to the CR tones have less space between them, there is a decrease in the response to treatment, with higher symptom scores during and after stimulation. Thus, higher gap indices—and thus loosely spaced CR tones—are associated with a more pronounced treatment response in the acute stage. These results hold true when controlling for both tinnitus duration and 5-PTA.

When examining VAS-L after regular aCR, only the duration was significant. When examining VAS-A after regular aCR, both gap index and duration were significant. The results of these exploratory analyses suggest that having had tinnitus for longer duration had a possible negative impact on therapeutic efficacy for the acute after-effect. In contrast, in a prospective, randomized, single-blind, placebo-controlled 12-week proof of concept study in 63 patients with chronic tonal tinnitus, up to 50 dB hearing loss and tinnitus duration characteristics comparable to the sample considered here, it was shown that tinnitus duration was not a confounding factor for patients treated with aCR stimulation for 4–6 hours per day (Tass et al., 2012a). Hence, while the acute effects studied in this paper may depend on tinnitus duration, long-term effects of aCR stimulation may evolve irrespective of tinnitus duration. The results of this study are applicable specifically to the acute effects of aCR during short, 16-min epochs. With these results in hand, it will be important to assess whether loosely spaced CR tones will also be favorable in a chronic stimulation setting, with the goal of inducing plastic changes furthering sustained long-term desynchronization and corresponding symptom relief. With regards to the other indices considered—mean relative gap and mean relative overlap—there were fewer models in which the VAS scores were explained solely by those variables, as summarized in Supplementary Table 6.

Of note, acute desynchronizing effects (obtained during stimulation) are not necessarily predictive of long-term desynchronizing effects emerging after cessation of stimulation. For instance, a theoretical study in spiking neuronal networks with STDP receiving periodic stimulation revealed complex and counterintuitive stimulus-response relationships between acute (de)synchronization during stimulation and sustained, long-lasting (de)synchronization emerging as time evolves after cessation of stimulation (Kromer and Tass, 2020). In particular, synchronization during stimulation may be followed by long-lasting desynchronization, and desynchronization during stimulation may end up in long-lasting synchronization. From a computational standpoint, acute desynchronizing effects of CR stimulation are favorable but not predictive of long-term desynchronization (Manos et al., 2018; Kromer and Tass, 2020), for which reason the gap index is not necessarily predictive of long-term effects.

There are some important differences between regular and noisy aCR that are worth mentioning. First, regular and noisy aCR differ slightly with respect to acute after-effects. In the original Adamchic et al., 2017 study, while both stimulation protocols caused significant after-effects on tinnitus loudness, only noisy aCR led to a significant reduction of tinnitus annoyance after the end of stimulation. From an electrophysiologic standpoint, regular aCR caused a significantly longer and stronger decrease of the delta band power, longer and stronger increase of alpha band power, and significantly longer decrease of gamma band power (Adamchic et al., 2017). However, interestingly in the window of 80–90 s after stimulation, the reduction in gamma band power is superior after noisy aCR. There is evidence to suggest that the gamma band is more pertinent to the loudness of the tinnitus percept (Van Der Loo et al., 2009), so once again, this may be why only noisy aCR showed a statistically significant acute after-effect in VAS-L after therapy, while regular aCR does not (and hence also why the gap index is only a significant predictor for VAS-L after noisy aCR).

These electrophysiologic differences suggest a likely underlying mechanistic difference. The two protocols also differ with respect to long-term effects. While noisy aCR does have an acute on-effect, this effect is not long-lasting. After cessation of stimulation, only regular aCR has a lasting off-simulation effect, while noisy aCR does not (Tass et al., 2012a). Because regular and noisy aCR demonstrate marked differences in the duration of effect as well as in electrophysiologic changes induced, it stands to reason that the underlying mechanism of the two protocols may be different. For example, as described in (Adamchic et al., 2017), the reduction of delta band power after the end of regular aCR was more pronounced and lasted longer than that after noisy aCR. Noisy aCR may behave similar to a masker, suppressing the tinnitus via feed-forward inhibition rather than strictly via long-lasting desynchronization (Roberts, 2007), though this is not known. Despite the possible differences in underlying mechanism between regular and noisy aCR, we found in this study that wider ERB spacing was found to be beneficial for both regular and noisy aCR. The reason for this is yet unknown. One possibility is that wider ERB spacing in noisy aCR leads to a greater degree of masking. As shown in Supplementary Figure 5, the mean relative overlap of CR tones 2 and 3 is negative for noisy aCR, indicating greater spacing. Evidence suggests that masking may be most efficient for sounds of frequency just below the tinnitus frequency (Terry et al., 1983). With a higher degree of spacing between tones, it may be that the functional spectrum of noisy aCR is wider on average, thus possibly contributing to greater residual inhibition, but this is speculative and requires further investigation. Furthermore, as was seen in (Tass et al., 2012a), the pitch of patients’ tinnitus tended to shift downward over the course of therapy in regular aCR. One possible explanation for this may be evident from the relative gaps. As seen in Supplementary Figure 1, in regular aCR, the pairwise relative gaps increase with increasing tone frequency in regular aCR (i.e., in (A), G (1,2)< G(2,3)<G(3,4)  on average), while in noisy aCR this is not true (i.e., in (B), G (1,2)< G(3,4)<G(2,3) on average). This may contribute to the lowering of the tinnitus pitch with therapy which has been observed with regular aCR only, on account of a possible downshifting of the tinnitus focus due to disproportionately effective desynchronization at higher stimulus frequencies in regular aCR.

Additional studies are required to validate the findings of this acute effects clinical study. To that end, there are several important considerations discussed herein that impact the interpretation of these results and the design of future experiments. Firstly, the ERB model itself utilizes several assumptions, which may limit the generalizability of this approach to specific subgroups of tinnitus patients. Secondly, various cochlear nonlinearities can impact the frequency specificity and thus the frequency-to-place match of auditory tonotopy which are unaccounted for in the existing ERB-transformation of CR tones. Thirdly, peripheral estimations of frequency selectivity are reasonable but imperfect representations of cortical tuning, i.e., the ERB model represents the behavior at the level of the basilar membrane more closely than it may represent the auditory cortex in full, which is more difficult to probe non-invasively. And finally, ascertaining an accurate pitch match is a necessity of aCR in order to determine the correct *f*_*T*_ around which to place the therapeutic tones, whether using fixed spacing or ERB-based spacing. Each of these considerations is discussed in detail in the Supplementary Discussion.

### Limitations of the ERB Model as Applied to Tinnitus

Auditory filter widths tend to broaden in individuals with cochlear deficits (Florentine et al., 1980; Glasberg and Moore, 1986), with the bandwidth typically increasing with increasing threshold. As described in Eq. 2, the ERB widths for hearing-impaired listeners can be estimated as a function of threshold at a given center frequency. However, there are some limitations to this approach. Firstly, this relationship does not hold for thresholds greater than 50 dB HL (Moore et al., 1999). Secondly, the relationship between ERB width and center frequency is best established for frequencies from 2,000 to 6,000 Hz (Moore et al., 1999), thus it is harder to comment on relationships outside of this spectral range. The tinnitus frequency in this cohort ranged from 675 to 9,800 Hz. Of the 36 ears across 18 patients, seven ears had a tinnitus pitch match between 2,000 and 6,000 Hz.

Notably, there is a weak correlation between threshold elevation on audiometry and the widening of the filter bandwidth, as there is variability on an individual level in the degree of broadening and in the asymmetry of the filter, possibly due to varying patterns of cochlear damage (Moore, 1995). This points to a need for measuring the ERB directly rather than estimating it from the absolute threshold, as was done in Eq. 2, in order to attain truly perceptually relevant tone spacing for aCR in any given individual.

Furthermore, the analysis was done on previously acquired data. As a result, it was not possible to manipulate the spacing of CR tones independently of the hearing thresholds, for which reason the 5-PTA demonstrated multicollinearity, i.e., high intercorrelation, with the gap index, mean relative gap, and mean relative overlap. In each of these multiple linear regressions, the 5-PTA variable was residualized to help reduce the effect of multicollinearity, as described in *Primary Analysis*. This involves regressing the two predictor variables and using the resulting residual variance in the overall multiple linear regression instead of one of the original predictors. However, this is a statistical estimation of the residual variance of the 5-PTA, and as a result, it is not possible within the framework of this re-analysis to definitively eliminate the relationship of hearing loss to each of these measures of tone spacing. In the future, additional experiments would require a within-subject approach to vary the tone spacing on the frequency axis (rather than the estimated ERB axis alone) while holding the underlying hearing thresholds constant in order to eliminate the effect of hearing loss on the ERB spacing arrangements. Ultimately, on account of these limitations, direct measurements of auditory filters may be required in individual patients rather than estimations based on tinnitus pitch and hearing thresholds. In the Supplementary Methods, we describe several methods by which the ERB widths may be directly measured in future studies. Additionally, in the Supplementary Discussion, we describe in further detail several other limitations and considerations unique to the ERB model, as introduced in *Overall Findings*.

### Recommendations for Future Studies

Based on the results of this study, we hypothesize that a larger gap index—and thus more loosely spaced CR tones—may improve the ability of tinnitus patients to respond positively to aCR acutely. Follow-up studies would involve the direct measurement of the ERBs of the auditory filters centered at the therapeutic tone frequencies, such as with one or more of the “rapid” methods described above. A first experiment may involve traditional, fixed spacing aCR compared head-to-head with aCR with some specified larger gap index between the ERBs of the therapeutic tones, on both an acute and chronic stimulation basis. Furthermore, our study at present was limited to the analysis of self-reported questionnaire data. Subsequent experiments should consider electrophysiologic measures of desynchronization as well, to assess the power changes that may result from different ERB-based aCR paradigms.

As a result of the afore mentioned cochlear nonlinearities and the related effect of stimulus level used to probe the auditory system’s frequency selectivity, it is evident that the desynchronization ability of individual CR tones may depend on more than simply the degree of overlap or gap between the corresponding ERBs. Upward and/or downward spread of masking, as well as lateral suppression, may have additional effects on CR efficacy, dependent on the magnitude and perhaps directionality of the individual inter-tone effects. As an example, if in fact there is pathologic downward spread of masking in SNHL, then higher frequency tones above *f*_*T*_ may themselves to some extent mask the tinnitus frequency. High-frequency CR tones may in theory also decrease the efficacy of lower-frequency CR tones. The inverse could be seen in excess upward spread of masking. To parse out these effects, follow-up experiments in tinnitus subjects may involve pairs of tones above and below the tinnitus frequency. The distance between the CR tones themselves and the distance between the CR tones and *f*_*T*_ could be varied systematically in order to determine degree of subjective tinnitus relief as well as degree of electrophysiologic desynchronization resulting from various spacing configurations. Considering as well that CR tones may be susceptible to the effects of forward masking, in which a probe tone is masked by a preceding masker tone, the temporal spacing of CR—in addition to frequency spacing—may also be an avenue of further exploration.

It should be noted that when reversing the residualization procedure in the methods, such that the residual variance of the gap index is calculated toward the 5-PTA, the model yields the same adjusted *R* ^2^ and *p*-value as the original analysis, as shown in Supplementary Table 3. However, the only significant predictor variable now is the 5-PTA, rather than the residual variance of the gap index. From this, one could ask whether the hearing loss is the true predictor variable of the effect on VAS-L during aCR, rather than the tone spacing driving the effect. However, as shown in Figure 3A, the gap index does not simply depend on the average hearing impairment around $f_{T}$ . The gap index strongly depends on shape of the audiogram around $f_{T}.$ To that end, several studies will be necessary in further disentangling the impact of hearing loss versus the impact of tone spacing on clinical outcome.

As shown in Figure 3A, the largest values of the gap index are, on average, for normal hearing, and the gap span (or the range of gap values dependent on the hearing impairment) increases with increasing *f*_*T*_. If normal hearing is more favorable, rather than inherently larger gap indices, we could provide a normal hearing subject with a relatively high *f*_*T*_ *,* such as on the order of 8 kHz, regular CR tones (in which the predicted gap index would be low for this patient). If the acute effect is favorable with significant tinnitus suppression during therapy, it may be that the absence of hearing impairment, rather than the tone spacing, is associated with clinical improvement. Further, if we were to provide wider tone spacing to this subject (essentially the spacing one would expect for an individual with moderate hearing loss) and the patient does not have clinical benefit, again this would suggest that the absence of hearing impairment is associated with improved outcomes, rather than loosely spaced tones on the ERB scale. The same effect should be observable throughout the *f*_*T*_ range. However, due to the fact that the gap span increases with increasing *f*_*T*_, we hypothesize that the difference between regular CR tone spacing and the mimicked hearing impairment spacing would be greatest when *f*_*T*_ is on the higher end of the spectrum (such as in the aforementioned hypothetical patient with an *f*_*T*_ of 8 kHz).

In an additional related experiment, one could test a patient with moderate hearing loss. With hearing loss, the gap index tends to decrease. To compensate for hearing impairment, we could increase the spacing and thereby the gap index. If the patient has a clinical reduction in tinnitus loudness during CR, this could mean the tone spacing is the more salient predictor than the hearing loss. In each of these cases, whether normal hearing or moderate hearing loss, we could provide the aCR tone spacing expected in the opposite scenario to differentiate the effects of hearing threshold and tone spacing. Furthermore, it would be of immense utility to test patients whose audiograms demonstrate hearing loss above the *f*_*T*_, hearing loss below the *f*_*T*_, and hearing loss in the vicinity of *f*_*T,*_ thereby directly measuring outcomes with differently shaped audiograms, as was done in Figure 3A for the 26 dB hearing loss.

Overall, the results of this study are a first step in the clinical optimization of tone spacing for aCR in order to obtain maximal anti-kindling and long-term therapeutic benefit. Broader spatial stimulation of CR tones, as indicated by higher gap indices, may result in improved relief from tinnitus loudness and annoyance in the acute stage of therapy. Additional studies are required to determine electrophysiologic correlation of these findings, to assess the impacts of chronic stimulation, and to correlate measured rather than estimated auditory filter widths with symptom and electrophysiologic data.

## Data Availability

The original contributions presented in the study are included in the article/Supplementary Material, further inquiries can be directed to the corresponding author.
