# Supplementary figures and images for: Treatment Tone Spacing and Acute Effects of Acoustic Coordinated Reset Stimulation in Tinnitus Patients
Source: Front Netw Physiol. 2021 Oct 6;1:734344. doi: 10.3389/fnetp.2021.734344 (PMC10012992; doi:10.3389/fnetp.2021.734344)

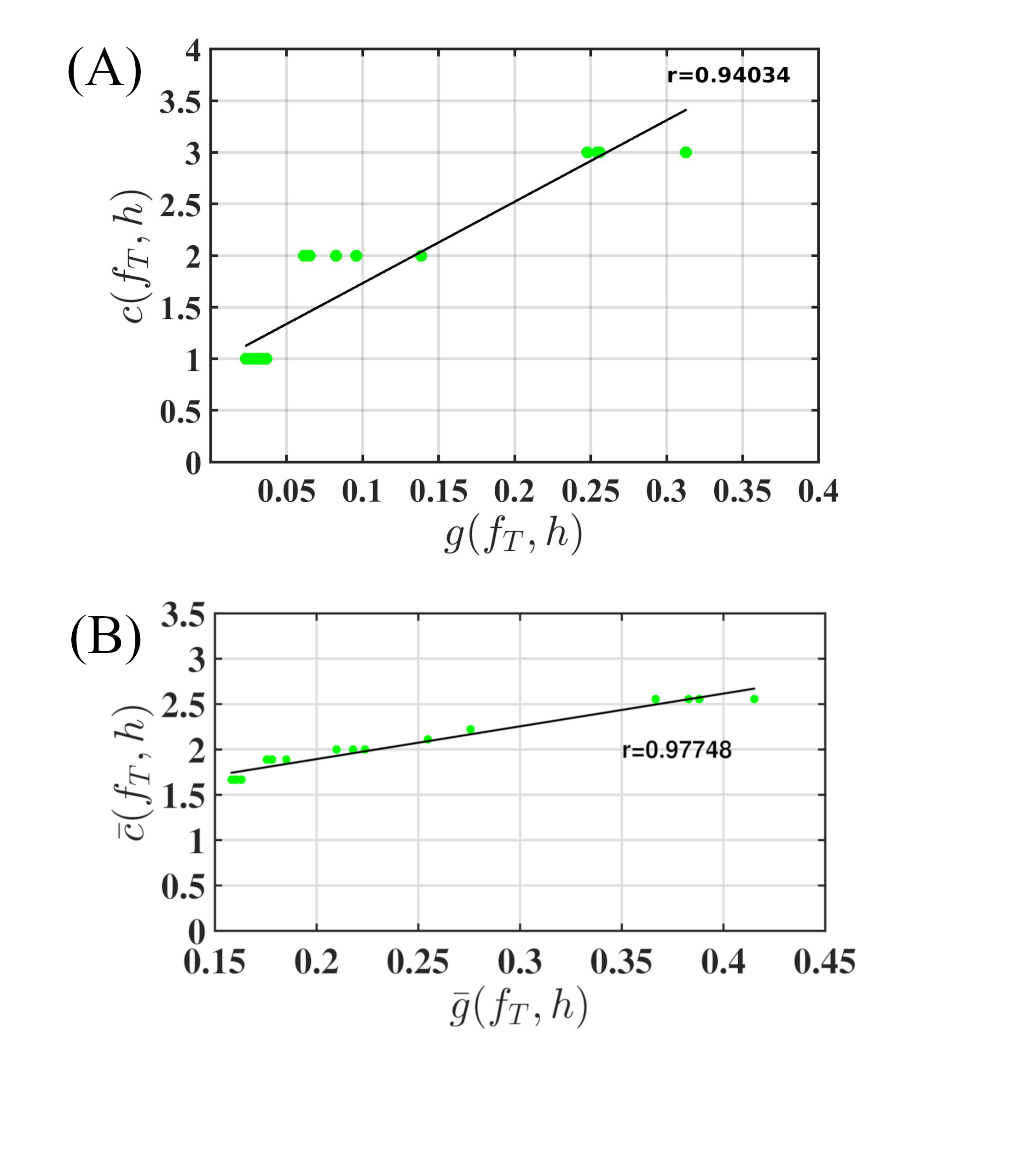

Supplement: Supplementary file 1 [file Image3.JPEG]

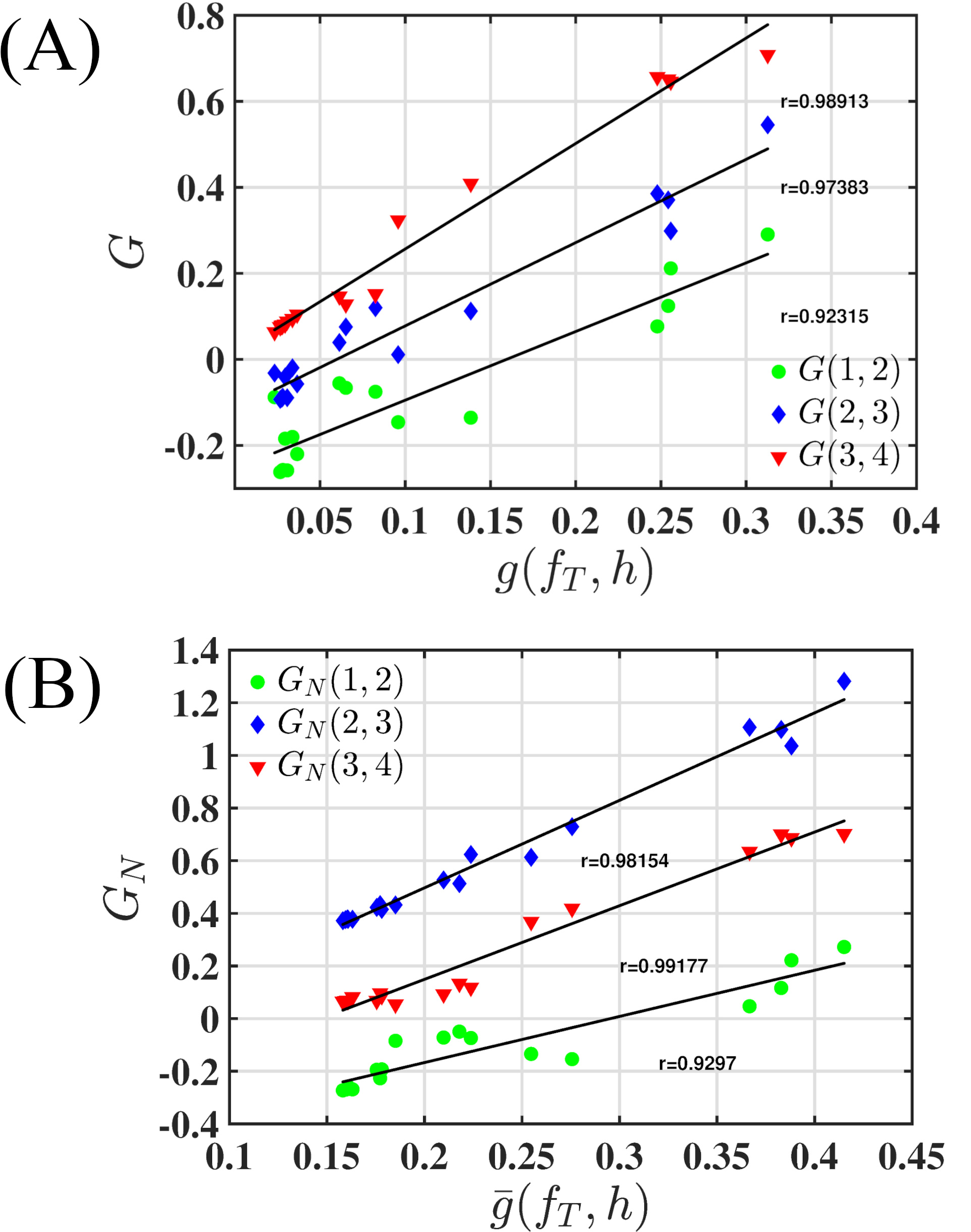

Supplement: Supplementary file 2 [file Image1.JPEG]

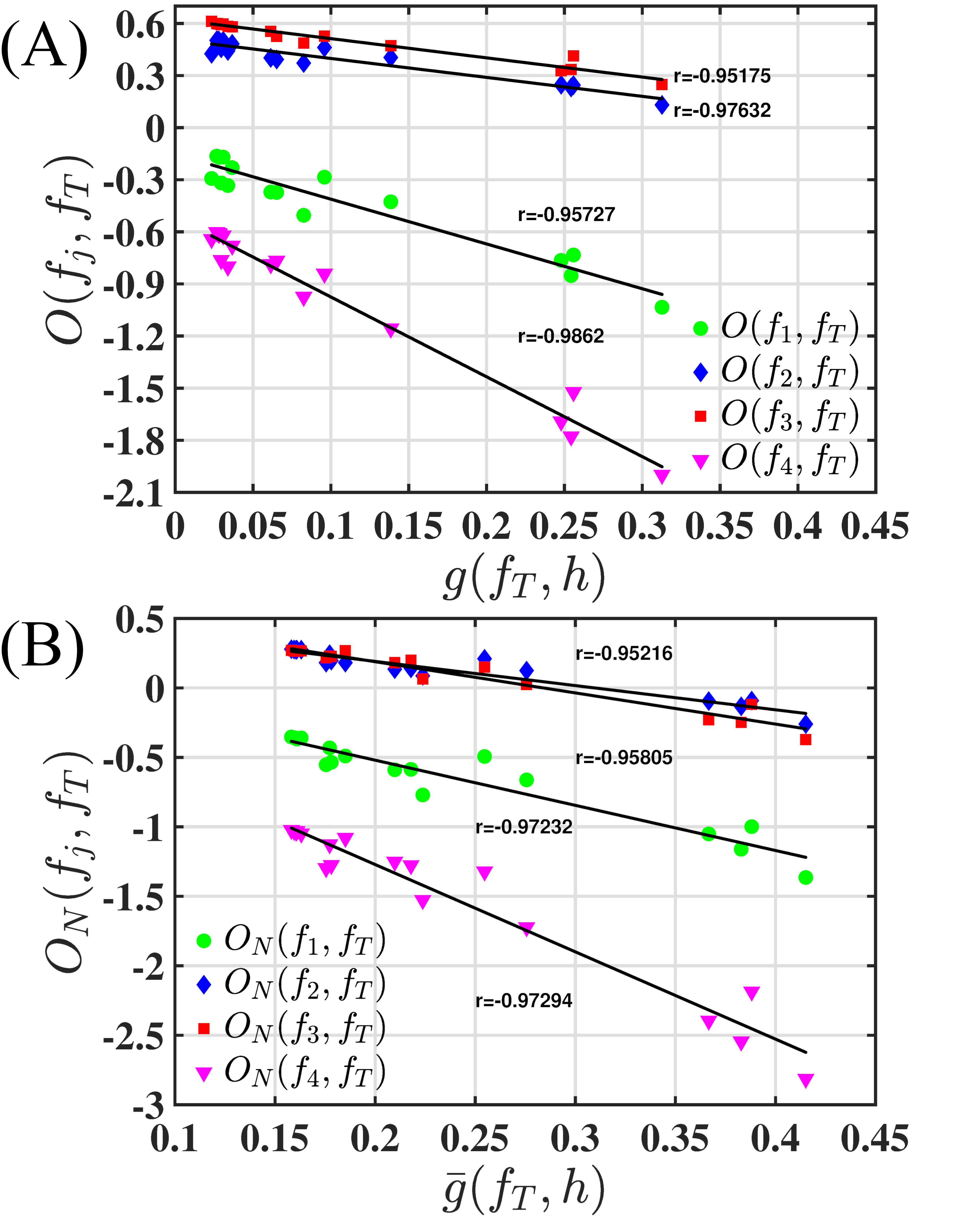

Supplement: Supplementary file 3 [file Image4.JPEG]

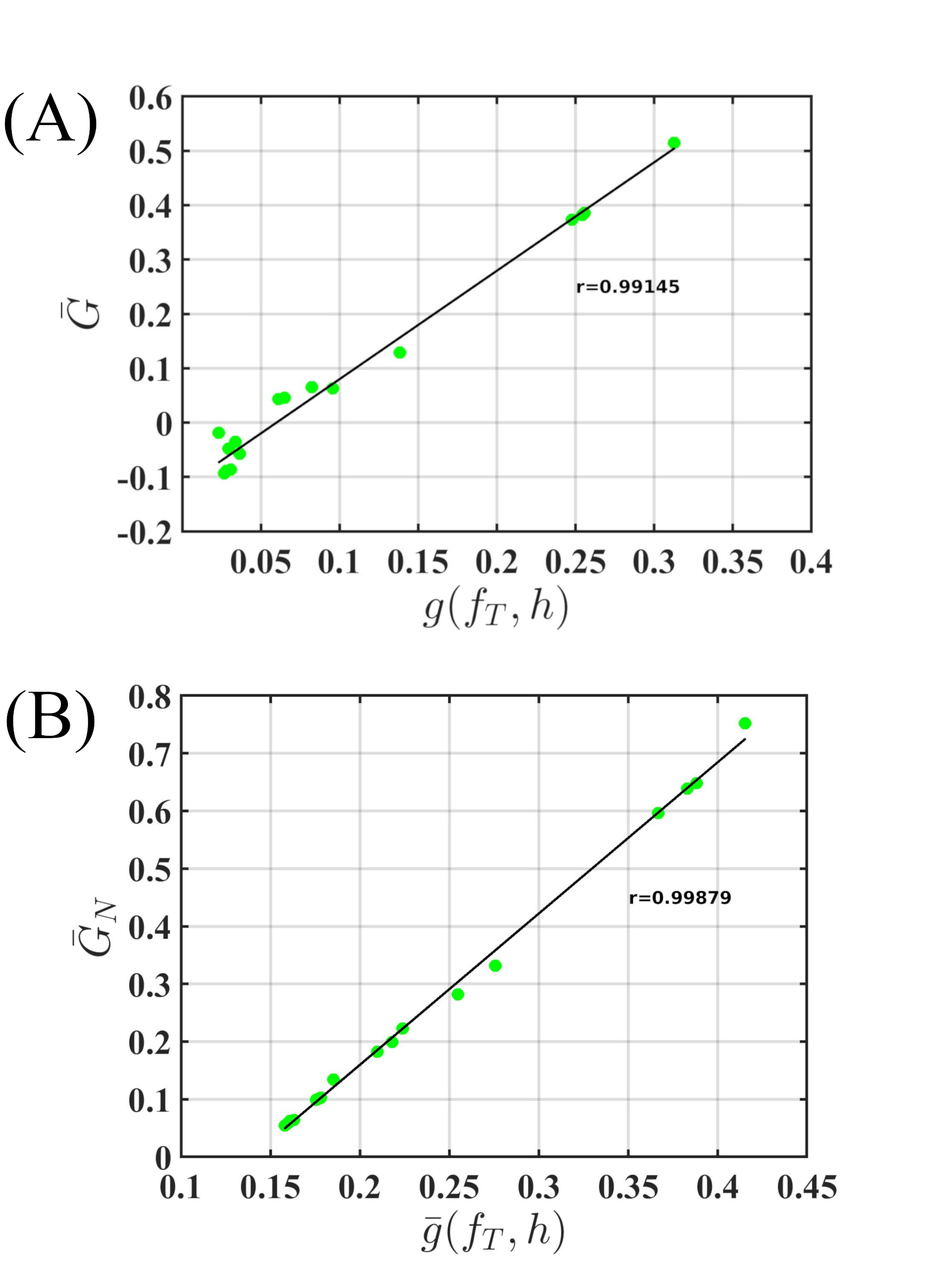

Supplement: Supplementary file 4 [file Image2.JPEG]

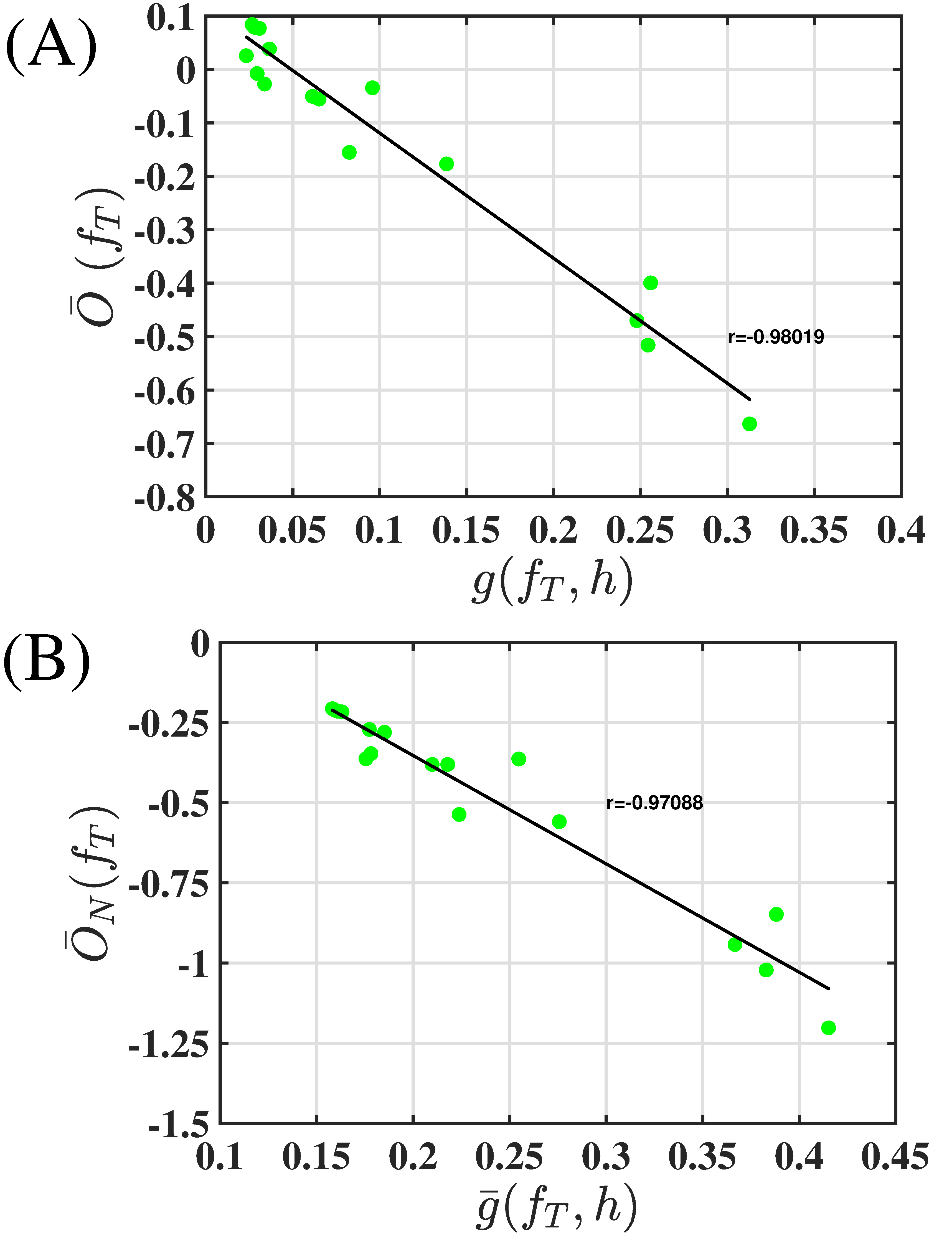

Supplement: Supplementary file 5 [file Image5.JPEG]
