# Supplementary material for: Treatment Tone Spacing and Acute Effects of Acoustic Coordinated Reset Stimulation in Tinnitus Patients
Source: Front Netw Physiol. 2021 Oct 6;1:734344. doi: 10.3389/fnetp.2021.734344 (PMC10012992; doi:10.3389/fnetp.2021.734344)
Supplement: Supplementary file 6 [file DataSheet1.docx]

Supplementary Material

**Supplementary Methods**

**Impact of shape of audiogram in the vicinity of tinnitus frequency** $\boldsymbol{f}_{\boldsymbol{T}}$ **on gap index**

In the main manuscript, we describe in Section 2.6 the impact of the shape of the audiogram on the gap index for non-homogenous hearing loss when the average hearing loss is 26 dB. Here, we also describe the case of hearing loss averaging 16 dB and 41 dB.

*On average 16dB hearing loss:*

For $\bar{h}\left( f_{T} \right)=16$ dB we consider three different cases with focal (model) hearing impairment:

(i) Lower-frequency hearing loss: $h\left( f_{1} \right)=50$ dB, $h\left( f_{2} \right)=14$ dB, $h\left( f_{3} \right)=h\left( f_{4} \right)=$ 0 dB.

(ii) Higher-frequency hearing loss: $h\left( f_{4} \right)=50$ dB, $h\left( f_{3} \right)=14$ dB, $h\left( f_{1} \right)=h\left( f_{2} \right)=$ 0 dB.

(iii) Tinnitus-centered hearing loss: $h\left( f_{3} \right)=50$ dB, $h\left( f_{2} \right)=14$ dB, $h\left( f_{1} \right)=h\left( f_{4} \right)=$ 0 dB.

Below 500 Hz, the gap index for higher-frequency (model) hearing impairment for $\bar{h}\left( f_{T} \right)=16$ dB approaches the gap index for homogenous hearing impairment for normal hearing $\bar{h}\left( f_{T} \right)=0$ dB (**Figure 3A**). On the other hand, up to 500 Hz the gap index for lower-frequency (model) hearing impairment for $\bar{h}\left( f_{T} \right)=16$ dB coincides with the gap index for homogenous hearing impairment for $\bar{h}\left( f_{T} \right)=26$ dB.

*On average 41 dB hearing loss:*

For $\bar{h}\left( f_{T} \right)=41$ dB we consider three different cases with (model) hearing impairment:

(i) Lower-frequency hearing loss: $h\left( f_{1} \right)=h\left( f_{2} \right)=h\left( f_{3} \right)=50$ dB, $h\left( f_{4} \right)=14$ dB.

(ii) Higher-frequency hearing loss: $h\left( f_{2} \right)=h\left( f_{3} \right)=h\left( f_{4} \right)=50$ dB, $h\left( f_{1} \right)=14$ dB.

The gap index for higher-frequency (model) hearing impairment for $\bar{h}\left( f_{T} \right)=41$ dB coincides with the gap index for homogenous hearing impairment for $\bar{h}\left( f_{T} \right)=50$ dB (not shown). Conversely, the gap index for lower-frequency (model) hearing impairment for $\bar{h}\left( f_{T} \right)=41$ dB approaches the gap index for homogenous hearing impairment for $\bar{h}\left( f_{T} \right)=26$ dB.

**Supplementary Discussion**

**Direct measurements of auditory filters**

Ultimately, on account of the limitations of the mathematical estimation of ERB widths as discussed in the main text, direct measurements of auditory filters may be required in individual patients rather than estimations based on tinnitus pitch and hearing thresholds. For this reason, we describe herein additional experiments and factors to consider when measuring auditory filter widths. Auditory filter width is typically determined through various masking experiments. In audiologic terms, masking refers to one sound, called a masker, modifying the threshold, or audibility, of another sound, called the probe (1960). The effect of masking is strongest when the frequency content of masker and probe is similar (Mayer, 1894;Wegel and Lane, 1924). Thus, masking can be used to gauge the frequency resolving abilities of the auditory system. The shape and width of the auditory filter can generally be derived experimentally through two methods: psychophysical tuning curves (PTCs) and auditory filters from notched-noise. Subsequently, the auditory filter derived from either method can then be quantified with an empirically determined ERB width.

Within psychophysical tuning curves, there are several experimental paradigms. Probe and masker can be presented at the same time in simultaneous masking, or asynchronously in forward (masker followed by probe) and backward masking (probe followed by masker) (Moore, 2012). In the simplest case of a psychophysical tuning curve obtained through simultaneous masking, a pure tone probe is presented with a masker consisting of either a second pure tone or, more preferably to avoid beat detection, narrowband noise. The level of masker needed to make the target inaudible is deemed the masked threshold. This is repeated with different masker center frequencies. This method is prone to off-frequency listening, since subjects can attend to more than one filter at a time, which causes the tip of the PTC to be sharper than if one filter could be isolated (Johnson‐Davies and Patterson, 1979;O'Loughlin and Moore, 1981;Moore, 2012). The second method of auditory filter estimation, referred to as notched-noise, is less prone to off-frequency listening (Patterson, 1976). A signal is fixed in frequency, and in this case, the masker consists of noise with a notch centered at the frequency of the signal. The threshold of the signal is determined as a function of the width of the notch. This typically results in a rounder top than the tip of the PTC due to a reduction in off-frequency listening (Moore, 2012).

Of note, single unit recordings in humans demonstrate that the frequency tuning of single neurons may actually exceed that of the auditory periphery (Bitterman et al., 2008), which would indicate that psychophysical tuning curves may underestimate the specificity of the cortex. Furthermore, in a study of otoacoustic emissions in humans, cochlear tuning at low sound levels (defined as 40 dB SPL) was found to be twice as sharp as determined by OAEs than by the corresponding PTCs (Shera et al., 2002). Thus, while psychophysical tuning curves can only be understood within the experimental bounds used to produce them, including stimulus rate and intensity, they serve as a reasonable noninvasive estimate that can be obtained in clinical and research settings. It should be noted that both PTC and notched-noise filter methods of auditory filter determination were developed in individuals with normal hearing. While subsequent experiments have determined that the filter shape and width change with cochlear lesions and hearing loss as described above, there is limited literature with regards to changes in the auditory filter that are specific to tinnitus (Penner, 1980). Ultimately, it is also important to acknowledge that psychophysical tuning curves are behavioral estimates of cochlear tuning, and even OAEs are indirect measurements (since the basilar membrane or auditory nerve cannot directly be assessed in live human subjects). As described by Shera et al. (2002), neural processing in the central nervous system can have an impact on psychophysical tuning curves to an unknown extent. Furthermore, even experience with tonal languages (Liu et al., 2020) and musical training (Bidelman et al., 2016) can impact these psychophysical tests.

While tinnitus is often accompanied by hearing loss, and thus one may expect the general deafferentation to affect the filter widths in a way similar to that seen in hearing loss alone, there is limited literature on whether auditory filters differ in patients suffering from tinnitus and hearing loss as opposed to hearing loss alone (particularly if tinnitus patients for any reason perform differently on masking experiments around the tinnitus pitch). McKee and Stephens used the notched-noise method to examine the auditory filter in tinnitus subjects with normal thresholds up to 8000 Hz and tinnitus-free controls (McKee and Stephens, 1992). The only center frequency probed was 2000 Hz, with a masker that had either no notch or a 1200 Hz-wide notch. The authors report that the frequency resolution was similar in the two groups. However, the *f_T_* of the tinnitus subjects was unknown, and thus the relationship of the *f_T_* to the 2000 Hz target cannot be quantified. In another study, Mitchell and Creedon (1995) similarly obtained PTCs in tinnitus subjects with normal thresholds up to 8000 Hz and tinnitus-free controls. The PTCs in each subject were obtained in the region of the tinnitus pitch match. Tinnitus subjects were found to have hypersensitive, or "splayed" tails, with occasionally elevated tips. However, a tone-on-tone masking paradigm was used, introducing the possibility of beats and off-frequency listening. No statistical analysis was performed on the Q_10_ measures (an estimate of bandwidth similar to the ERB) between groups. Considering the limitations of these studies and their potentially contradictory results, with one reporting no change in filter tuning in tinnitus subjects and the other reporting a significant change in tinnitus subjects, it is evident that further research is needed specifically to examine the relationship of tinnitus to the frequency tuning of the auditory system, as well as possible differences secondary to challenges in executing the task which may be due to competing attention given to the tinnitus percept.

Ultimately, estimations of filter width based mathematically on center frequency and hearing threshold (as in Equation (2)) are but estimates. However, the methods to obtain both the PTC and the notched-noise auditory filter shape empirically are limited severely by the time that these procedures take. Depending on the number of filters assessed, this can take several hours and possibly even more than one day of testing. Several studies are exploring modified ways to obtain estimates of the width and shape of the auditory filter. Some recent advancements in this area are described herein and may be considered as a foundation for further experiments.

In the realm of notched-noise, Nakaichi et al. (2003) reported being able to estimate the auditory filter shape from a single masked threshold. In this, the two-alternative forced choice methodology is replaced with an ascending method. The time required for the procedure is estimated at two minutes per filter. The software used in this paper is not readily available and could require advanced coding for implementation. Another example of rapid estimation of frequency selectivity with the notched-noise method was proposed by Shen and Richards (2013), which uses an adaptive parameter-estimation procedure called the “quickAF” or “qAF”, replacing the traditional, more time-consuming threshold-based procedure. However, as with prior descriptions of the impact of hearing loss on ERB width (Moore et al., 1999), the authors indicate that the qAF procedure may only be sufficient in describing the auditory filter shape in individuals with thresholds less than 50 dB HL at the tested frequency (Shen et al., 2019).

In the realm of PTCs, a notable example of rapid measurement comes in the form of swept-noise PTC, or SWPTC (Sęk et al., 2005;Sęk et al., 2007;Sęk and Moore, 2011). This method makes use of Bekesy tracking. The masker is a band of noise that is swept in center frequency. The signal is pulsed on and off in the presence of the continuous masker. The subject then indicates when the stimulus is detectable. The shapes of the PTCs measured with SWPTC compared to those measured with traditional alternative-forced choice methods were found to be similar in both normal hearing and hearing-impaired subjects (Sęk et al., 2005;Sęk et al., 2007). The tuning estimates are also similar to those produced by traditional notched-noise methods, and the results are also unaffected by training (Charaziak et al., 2012). The time required is estimated at three to four minutes per filter. SWPTC software is publicly available and can be executed on a standard PC with no advanced coding needed. Konadath and Puttabasappa (2016) utilized the SWPTC method in tinnitus subjects and tinnitus-free controls. They found no change in the Q_10_ (similar to an ERB) between groups but did find a shift in tip frequency in tinnitus subjects. Further work is needed to elucidate the differences in frequency selectivity in tinnitus subjects and tinnitus-free controls. These “rapid” measurements of tuning may be an efficient avenue by which to obtain additional data and facilitate the testing of various acoustic CR spacing patterns based on the ERB model in individuals with tinnitus.

**The impact of cochlear nonlinearities and stimulus level on frequency selectivity**

Although the ERB model provides a reasonable estimate of frequency selectivity and thus spatial stimulation profiles, there are several key characteristics of the auditory system that can modify estimates of frequency selectivity in a manner that may be relevant to the provision of efficacious acoustic CR. In the auditory periphery, cochlear tuning can be affected by signal level, upward spread of masking, downward spread of masking, and lateral suppression. These factors, as well as the complex relationship between cochlear and cortical tuning, are described herein.

As discussed previously, individuals with hearing loss have decreased frequency selectivity compared to those with normal hearing. However, it is also known that frequency selectivity tends to decrease in normal hearing individuals with increasing signal level (Zwicker and Schorn, 1978). Since determinations of auditory filter width in individuals with hearing loss by necessity require higher signal levels due to their elevated thresholds in quiet, it is important to determine to what degree the widening of auditory filters in sensorineural hearing loss is pathologic and to what degree it is simply reflective of expected signal-dependent changes in the normal cochlea (Dubno and Schaefer, 1991).

A phenomenon of crucial consideration here is what is referred to as the upward spread of masking, in which higher frequencies are more susceptible to masking by lower frequencies (Wegel and Lane, 1924;Egan and Hake, 1950). This occurs during simultaneous masking, but in fact, the effect is largest in forward masking (Oxenham and Plack, 1997;Plack et al., 2002). This effect is level-dependent, such that higher masker levels lead to even more pronounced masking of the high frequencies. This nonlinear increase in masking at high frequencies is observed even in normal hearing ears. Several studies have suggested that ears with hearing loss are susceptible to excess masking beyond what would be seen in the physiologic state, contributing to the widening of the auditory filter and decreased frequency resolving capacity in individuals with hearing loss (Trees and Turner, 1986;Gagné, 1988).

In contrast to these findings, Nelson demonstrated that when accounting for sound level variations needed to obtain PTCs between normal hearing listeners and hearing impaired listeners, hearing impaired listeners did not have pathologic upward spread of masking (Nelson, 1991). Instead, there was evidence of another, less well understood phenomenon responsible for decreased frequency resolution in individuals with SNHL, referred to as downward spread of masking. In downward spread of masking, high frequency maskers more effectively mask low frequency probe tones. This downward spread of masking was most evident when hearing loss at the probe frequency exceeded 40 dB, with some inter-individual variation (Nelson, 1991). Thus, there remains some uncertainty in quantifying the degree of contribution from excess upward and/or downward spread of masking to the abnormal frequency selectivity seen in SNHL.

The opportunity to tune stimulation amplitude used in computational modeling was absent in the clinical study of acute effects (Adamchic et al., 2017). While it is known that higher stimulus levels tend to broaden auditory filter widths, the precise nature of this relationship is not fully elucidated. As a result, auditory filter widths reported in the literature are not typically “normalized” for the stimulus level—either masker or probe—used to generate them. This is an avenue for further research, as a concise metric of auditory filter width accounting for the underlying parameters would greatly facilitate the correlation of peripheral and central frequency-resolving capacity of the auditory system, as described in Section 4.3.

**Relationship between peripheral and central spatial stimulation**

Although the auditory system is tonotopically arranged from periphery to cortex, the frequency resolving capacities and functions of each locus are still somewhat distinct. Thus, it is important to know to which degree behavioral estimates of frequency-resolving ability, such as those determined by psychophysical tuning curves, reflect the response of cortical neurons. The ascending auditory pathway can be thought of as a “multiresolution analyzer” consisting of several sequential filters, each with its own bandwidth and intensity dependence (Schreiner et al., 2000), with the cortex serving as the final filter.

For this reason, measures of frequency selectivity at the periphery are but an estimate of the true resolution in the cortex, which is the ultimate target of CR-based therapies. Firstly, the level dependence of frequency selectivity in the auditory cortex is reduced as compared to in the periphery (Sutter, 2000;Sadagopan and Wang, 2008). In other words, while stimulus level may more significantly impact frequency resolution and thus estimates of filter width at the level of the cochlea, cortical frequency resolution may be less susceptible to the effects of stimulus amplitude in comparison. The spectrotemporal density of stimuli may be a key factor in determining this level tolerance at the cortical level. In a demonstrative experiment by Pienkowski and Eggermont, spike and local field potential (LFP) recordings were obtained from primary auditory cortex in anesthetized cat. Two sets of stimuli were delivered, one that was spectrotemporally sparse and another that was spectrotemporally dense. Within a seven-octave range, sparse stimuli were presented at 4/s while the dense stimuli were presented at 28/s. For sparse stimuli, increasing sound level resulted in more variable tuning properties of the individual unit and LFP responses. However, for the spectrotemporally dense stimuli, the tonotopic representation was more level tolerant, at least up to 65 dB SPL (Pienkowski and Eggermont, 2011). Thus, there is some degree of cortical compensation for the peripheral loss of frequency specificity with increasing sound level in spectrotemporally dense stimuli, as are typically heard in the natural environment. This may be a relevant feature for future exploration in acoustic CR.

Electroencephalographic (EEG) and magnetoencephalographic (MEG) findings in humans corroborate the relative level tolerance of the cortical tonotopic map. The N100 wave in EEG and N100m wave in MEG experiments are a proxy for psychophysically obtained measures of frequency selectivity. When two stimuli are closer together in frequency, the resulting N100 amplitude tends to be smaller, due to a decrease in responsiveness to the second tone. This is called frequency-specific adaptation (Jääskeläinen et al., 2007;Herrmann et al., 2013). In one study, subjects were presented with test tones of 1000 Hz in association with additional tones varying between 578 and 1728 Hz, with a constant interstimulus interval of 460 ms. The N100 amplitude was smaller for smaller frequency separations (Näätänen et al., 1988). In a similar study by Sams and Salmelin, a notched noise experiment in conjunction with MEG was performed, using 100-millisecond tones that were 1000 and 2000 Hz, with the interstimulus interval varying between 1s and 5s. The wider the notch in the masker, correlating to a wider peripheral auditory filter, the shorter the N100m latency and the larger the N100m amplitude (Sams and Salmelin, 1994). Thus, The N100 and N100m are an attractive target to further understand the level tolerance of the cortical tonotopic map. To that end, Herrmann et al. presented normal hearing individuals with stimuli with varying degrees of spectral variance, as well as two intensity levels, one soft at 35 dB SL and one loud at 60 dB SL. The frequency-specific adaptation, whereby a second stimulus close in frequency to the first elicits an N100 of smaller amplitude, was the same regardless of sound level, indicating level tolerance of the frequency-specific neural responses (Herrmann, 2013). This implies that the cortex may be compensating for the decreased frequency-resolving capacity of the cochlea through a nonspecific increase in central sensory gain (Herrmann, 2013).

Furthermore, it is important to note that there is a non-linear relationship between stimulus intensity and perceived loudness. Loudness is a subjective feature and depends on both the subject and the stimulus. Scharf defines four contributors to loudness, including stimulus intensity, spectral content, time, and background (Scharf, 1978). The most salient of these is stimulus intensity. At the auditory periphery, the vibration of sound traverses the basilar membrane. As the sound intensity is increased, the firing rate of the nerve fibers from within the stimulated bands increases, as does the area over which the stimulus traverses and thus the total number of nerve fibers responding to the stimulus (Schreiner and Malone, 2015). Sound energy within the stimulated critical bands is integrated with the Steven’s power law L = kI^0.3^, where L is the perceptual loudness, k is a subject-specific constant, and I is the sound intensity (Stevens, 1957;Scharf, 1978;Schreiner and Malone, 2015). However, as noted above, non-intensity factors play a role in perceived loudness and may also have an effect on the degree of cortical activation. Furthermore, in hearing impaired ears, there is an additional consideration called loudness recruitment, whereby there is an unusually rapid growth of loudness with increasing sensation level (Moore, 2012). These factors further obscure the relationship between stimulus intensity and frequency tuning. Thus, while psychophysical estimates of frequency tuning are not generally normalized for stimulus intensity and certainly not for perceived loudness, the relative intensity independence of the cortical frequency response suggests that the ERB concept may still be a valid approach to the optimization of acoustic CR tone spacing. The precise relationships between the peripheral ERB widths, stimulus amplitudes, and central tuning—and how each of these relationships may affect acoustic CR—remain to be fully elucidated.

**Pitch matching**

A final important consideration in the provision of acoustic CR is the ascertainment of the tinnitus frequency, since optimal provision of CR—regardless of tone spacing—ultimately requires a very accurate pitch match around which to center the therapeutic tones.

Generally, the tinnitus percept is subjectively described by patients with a variety of terms, including humming, buzzing, and ringing (Savastano, 2004;Lentz and He, 2020). Nevertheless, in many cases the tinnitus percept is either itself a pure tone (i.e., tonal tinnitus) or a pure tone with additional noise. A study of 205 cases of tinnitus in Japan found through that 61% of patients had noise-like tinnitus, while 39% of patients had tonal tinnitus (Kodama and Kitahara, 1990). In another cohort, 59% of subjects had tonal tinnitus, 25% had noise-like tinnitus, and 16% had a combination of the two (Turner, 1990). Thus, the relatively high estimates of the prevalence of tonal tinnitus indicate that acoustic CR may be beneficial to significant subsets of the population suffering from tinnitus.

Within tonal or combination tinnitus, there have been efforts to determine the prevalence of various possible center frequencies, which is particularly relevant considering the ERB model is most well understood in the frequency range of 2000 to 6000 Hz. Pitch matching involves, by various methodologies, asking patients to identify which stimulus tones are most similar in pitch to their tinnitus percept. One of the early attempts at elucidating the spectral content demonstrated that the prevalence of a central tinnitus frequency between 2000 Hz and 6000 Hz was 55% in a group of 200 individuals (Reed, 1960). However, subsequent studies have had different results. Later, in 1046 patients at the Tinnitus Clinic of the Oregon Health Sciences Center, the distribution of center frequency was as follows: 11% between 0 and 1000 Hz; 21% between 1001 and 3000 Hz; 30% between 3001 and 5000 Hz; 18% between 5001 and 7000 Hz; 14% between 7001 and 9000 Hz; 4% between 9001 and 11,000 Hz; and 1% between 11,001 and 15,000 Hz (Meikle and Taylor-Walsh, 1984). The largest study known to date was described by Savastano (2004), in which across 1440 subjects with tinnitus, the distribution was as follows: 27.6% between 0 and 1000 Hz; 10.3% between 1001 and 3000 Hz; 17.3% between 3001 and 6000 Hz; 10.3% between 6001 and 8000 Hz; and 31% >8000 Hz. Thus, 96.5% of subjects fell into one of these categories, with the remaining 3.5% of subjects having a tinnitus described as white noise.

The variability across all of these studies highlights the challenge of obtaining an accurate pitch match, which is a critical factor for many sound-based therapies including acoustic CR. Matching the pitch of tinnitus is a notoriously difficult task, prone to test-retest variability, pitch fluctuation, octave confusion, and the presence of multiple pitches in the percept (Penner and Bilger, 1989;Henry and Meikle, 2000;Tyler, 2000). The method utilized, the order in which stimuli are presented (either ascending or descending), and even the ear in which stimuli are presented can all have a major impact on the resulting pitch-matched frequency (Tyler and Conrad-Armes, 1983). There have been efforts put forth to improve and standardize the pitch matching procedure, including the use of Bayesian statistics (McMillan et al., 2014), but as of yet there is no single protocol in use in clinical and research settings. An accurate *f_T_* is of paramount importance in the ability of acoustic CR to result in therapeutic desynchronization, for which reason the execution of rigorous pitch matching prior to initiation of therapy is not trivial.

Furthermore, although the original acoustic CR approach was designed for patients with subjective tonal tinnitus (Tass et al., 2012a) rather than those with noise-like tinnitus, an appropriate detection of the tinnitus-involved frequency range may enable one to extend the concept of acoustic CR stimulation to non-tonal tinnitus. For instance, Norena et al. have proposed an alternate method by which to describe the frequency content of the tinnitus percept rather than a singular pitch match, called “internal tinnitus spectra,” which may better take into account the complexity of the tinnitus sensation (Norena et al., 2002). Such a measurement of the internal tinnitus spectra may be useful in determining and possibly even expanding candidacy for acoustic coordinated reset (CR) stimulation if one or a few tones are dominant within a given band of frequencies. The ERB model could subsequently be applied to transform each of the selected therapeutic tones into perceptually relevant spacing not just for pure tonal tinnitus, but possibly also for narrowband tinnitus.

**Supplementary Figure Legends**

**Supplementary Figure 1:** The relationships between (**A**) the relative gap $G$ and the gap index $g$ for each subject in regular acoustic CR and between (**B**) the relative gap $G_{N}$and the gap index $\bar{g}$for noisy acoustic CR. For both types of stimulation, the relative gap is highly correlated with the gap index, regardless of whether the relative gap is negative, zero, or positive. Notably, only for regular acoustic CR do the pairwise relative gaps increase with increasing tone frequency (i.e., in (**A**), $G \left( 1,2 \right)< G\left( 2,3 \right)<G\left( 3,4 \right)$on average, while in (**B**), $G_{N} \left( 1,2 \right)< G_{N}\left( 3,4 \right)<G_{N}\left( 2,3 \right)$. This may contribute to the lowering of the tinnitus pitch with therapy which has been observed with regular CR only, on account of a possible downshifting of the tinnitus focus due to disproportionately effective desynchronization at higher stimulus frequencies in regular CR.

**Supplementary Figure 2:** The mean relative gap $\bar{G}$ between all pairs of neighboring ERBs is also highly correlated with the gap index *g* in regular acoustic CR, as shown in (**A**). The mean relative gap $\bar{G}_{N}$ is highly correlated with the gap index $\bar{g}$in noisy acoustic CR, as shown in (**B**).

**Supplementary Figure 3:** As shown in (**A**), the gap count *c* is highly correlated with the gap index *g* for regular acoustic CR. In (B), the gap count $\bar{c}$ is highly correlated with the gap index $\bar{g}$in noisy acoustic CR.

**Supplementary Figure 4**: The relationships between the relative overlap *O* (quantifying the overlap of each tone’s ERB with the tinnitus ERB) and the gap index *g* for each subject in (**A**) regular acoustic CR and (**B**) between $O_{N}$ and $\bar{g}$ in noisy acoustic CR. For both types of stimulation, the relative overlap is highly correlated with the gap index, regardless of whether the relative overlap is negative, zero, or positive. This indicates that the gap index is inclusive of relationships of therapeutic tone ERBs to the tinnitus ERB. Furthermore, for regular CR, even at large values of the gap index, there is overlap of the ERBs for tones 2 and 3 with the tinnitus ERB. In noisy CR, however, the ERBs for tones 2 and 3 do not overlap with the tinnitus ERB. This may be relevant in the different mechanisms for the two therapeutic paradigms.

**Supplementary Figure 5:** (**A**) The mean relative overlap $\bar{O}$ between all therapeutic tones and the tinnitus ERB is also highly correlated with the gap index g in regular acoustic CR. (**B**) The mean relative overlap $\bar{O}_{N}$ is highly correlated with $\bar{g}$in noisy acoustic CR.

**Supplementary Tables**

**Supplementary Table 1:** Demographic and audiometric data for all participants. It should be noted that 14 subjects had bilaterally symmetric tinnitus. For these subjects, the tinnitus frequency was assessed in both ears separately and documented as such. For the four subjects with right-dominant tinnitus, only the right ear was tested. The tinnitus frequency for the left ear is thus noted as “N/A,” i.e., not applicable. Within the audiometric data, NR denotes “no response.” In these cases, the subject did not respond at the limits of the audiometer.

**Supplementary Table 2**: Normalized VAS-L and VAS-A scores during and after therapy for both regular and noisy acoustic CR are shown here.

**Supplementary Table 3:** Multiple linear regression model for VAS-L during therapy using tinnitus duration, residual variance of gap index to 5-PTA, and 5-PTA as predictor variables. In this case, reversing the residualization procedure leads to the same overall *R*^2^ and *p*-value as in the primary analysis. However, now the hearing loss is significant while the residual variance gap index is not. As shown in **Figure 3A**, however, the shape of the audiogram is of crucial importance in determining the gap index. Thus, the relationship of these variables is not 1:1 and cannot strictly be predicted by the value of the pure tone average. * denotes statistically significant result for alpha level <0.05.

**Supplementary** **Table 4:** Multiple linear regression models using tinnitus duration, mean relative gap, and residual variance of 5-PTA to mean relative gap as predictor variables. The outcome variables are VAS-L during and after therapy and VAS-A during and after therapy. Findings are shown for both regular aCR and noisy aCR. *B* (*SE*) represents unstandardized beta with standard error in parentheses. * denotes statistically significant result for alpha level <0.05, while † denotes trending towards significance.

**Supplementary Table 5:** Multiple linear regression models using tinnitus duration, mean relative overlap, and residual variance of 5-PTA to mean relative gap as predictor variables. The outcome variables are VAS-L during and after therapy and VAS-A during and after therapy. Findings are shown for both regular aCR and noisy aCR. *B* (*SE*) represents unstandardized beta with standard error in parentheses. * denotes statistically significant result for alpha level <0.05, while † denotes trending towards significance.

**Supplementary Table 6:** Summary of all linear regression models, with the primary analysis in bold. Each cell indicates which input variables were found to be significant predictors within each model.

**Supplementary References**

(1960). *Acoustical Terminology SI.* New York: American Standards Association Std.

Adamchic, I., Toth, T., Hauptmann, C., Walger, M., Langguth, B., Klingmann, I., and Tass, P.A. (2017). Acute effects and after-effects of acoustic coordinated reset neuromodulation in patients with chronic subjective tinnitus. *NeuroImage: Clinical* 15**,** 541-558.

Bidelman, G.M., Nelms, C., and Bhagat, S.P. (2016). Musical experience sharpens human cochlear tuning. *Hearing Research* 335**,** 40-46.

Bitterman, Y., Mukamel, R., Malach, R., Fried, I., and Nelken, I. (2008). Ultra-fine frequency tuning revealed in single neurons of human auditory cortex. *Nature* 451**,** 197-201.

Charaziak, K.K., Souza, P., and Siegel, J.H. (2012). Time-efficient measures of auditory frequency selectivity. *International Journal of Audiology* 51**,** 317-325.

Dubno, J.R., and Schaefer, A.B. (1991). Frequency selectivity for hearing-impaired and broadband-noise-masked normal listeners. *The Quarterly Journal of Experimental Psychology Section A* 43**,** 543-564.

Egan, J.P., and Hake, H.W. (1950). On the masking pattern of a simple auditory stimulus. *The Journal of the Acoustical Society of America* 22**,** 622-630.

Gagné, J.P. (1988). Excess masking among listeners with a sensorineural hearing loss. *The Journal of the Acoustical Society of America* 83**,** 2311-2321.

Henry, J.A., and Meikle, M.B. (2000). Psychoacoustic measures of tinnitus. *Journal of the American Academy of Audiology* 11**,** 138-155.

Herrmann, B., Henry M. J., Scharinger, M., Obleser J. (2013). Auditory filter width affects response magnitude but not frequency specificity in auditory cortex. *Hearing Research***,** 128-136.

Herrmann, B., Henry, M.J., and Obleser, J. (2013). Frequency-specific adaptation in human auditory cortex depends on the spectral variance in the acoustic stimulation. *Journal of neurophysiology* 109**,** 2086-2096.

Jääskeläinen, I.P., Ahveninen, J., Belliveau, J.W., Raij, T., and Sams, M. (2007). Short-term plasticity in auditory cognition. *Trends in neurosciences* 30**,** 653-661.

Johnson‐Davies, D., and Patterson, R.D. (1979). Psychophysical tuning curves: Restricting the listening band to the signal region. *The Journal of the Acoustical Society of America* 65**,** 765-770.

Kodama, A., and Kitahara, M. (1990). Clinical and audiological characteristics of tonal and noise tinnitus. *ORL* 52**,** 156-163.

Konadath, S., and Puttabasappa, M. (2016). Psychophysical Tuning Curves and Extended High Frequency Audiometry Findings in Individuals with Normal Hearing Having Tinnitus. 6**,** 371-378.

Lentz, J.J., and He, Y. (2020). Perceptual Dimensions Underlying Tinnitus-Like Sounds. *J Speech Lang Hear Res* 63**,** 3560-3566.

Liu, Y., Xu, R., and Gong, Q. (2020). Human Auditory-Frequency Tuning Is Sensitive to Tonal Language Experience. *Journal of Speech, Language, and Hearing Research* 63**,** 4277-4288.

Mayer, A.M. (1894). Researches in acoustics. *American Journal of Science***,** 1-28.

Mckee, G., and Stephens, S. (1992). An investigation of normally hearing subjects with tinnitus. *Audiology* 31**,** 313-317.

Mcmillan, G.P., Thielman, E.J., Wypych, K., and Henry, J.A. (2014). A Bayesian perspective on tinnitus pitch matching. *Ear and Hearing* 35**,** 687-694.

Meikle, M., and Taylor-Walsh, E. (1984). Characteristics Of Tinnitus And Related Observations In Over 1800 Tinnitus Clinic Patients. *The Journal of Laryngology, Rhinology, and Otology* 98**,** 17-21.

Mitchell, C.R., and Creedon, T.A. (1995). Psychophysical tuning curves in subjects with tinnitus suggest outer hair cell lesions. *Otolaryngology–Head and Neck Surgery* 113**,** 223-233.

Moore, B.C. (2012). *An introduction to the psychology of hearing.* Brill.

Moore, B.C.J., Vickers, D.A., Plack, C.J., and Oxenham, A.J. (1999). Inter-relationship between different psychoacoustic measures assumed to be related to the cochlear active mechanism. *The Journal of the Acoustical Society of America* 106**,** 2761-2778.

Näätänen, R., Sams, M., Alho, K., Paavilainen, P., Reinikainen, K., and Sokolov, E. (1988). Frequency and location specificify of the human vertex N1 wave. *Electroencephalography and clinical neurophysiology* 69**,** 523-531.

Nakaichi, T., Watanuki, K., and Sakamoto, S. (2003). A simplified measurement method of auditory filters for hearing-impaired listeners. *Acoustical science and technology* 24**,** 365-375.

Nelson, D.A. (1991). High-level psychophysical tuning curves: Forward masking in normal-hearing and hearing-impaired listeners. *Journal of Speech, Language, and Hearing Research* 34**,** 1233-1249.

Norena, A., Micheyl, C., Chéry-Croze, S., and Collet, L. (2002). Psychoacoustic characterization of the tinnitus spectrum: Implications for the underlying mechanisms of tinnitus. *Audiology and Neuro-Otology* 7**,** 358-369.

O'loughlin, B., and Moore, B. (1981). Improving psychoacoustical tuning curves. *Hearing Research* 5**,** 343-346.

Oxenham, A.J., and Plack, C.J. (1997). A behavioral measure of basilar-membrane nonlinearity in listeners with normal and impaired hearing. *The Journal of the Acoustical Society of America* 101**,** 3666-3675.

Patterson, R.D. (1976). Auditory filter shapes derived with noise stimuli. *The Journal of the Acoustical Society of America* 59**,** 640-654.

Penner, M. (1980). Two-tone forward masking patterns and tinnitus. *Journal of Speech, Language, and Hearing Research* 23**,** 779-786.

Penner, M., and Bilger, R.C. (1989). Adaptation and the masking of tinnitus. *Journal of Speech, Language, and Hearing Research* 32**,** 339-346.

Pienkowski, M., and Eggermont, J.J. (2011). Cortical tonotopic map plasticity and behavior. *Neuroscience & Biobehavioral Reviews* 35**,** 2117-2128.

Plack, C.J., Oxenham, A.J., and Drga, V. (2002). Linear and nonlinear processes in temporal masking. *Acta acustica united with acustica* 88**,** 348-358.

Reed, G.F. (1960). An Audiometric Study of Two Hundred Cases of Subjective Tinnitus. *A.M.A. Archives of Otolaryngology* 71**,** 84-94.

Sadagopan, S., and Wang, X. (2008). Level invariant representation of sounds by populations of neurons in primary auditory cortex. *Journal of Neuroscience* 28**,** 3415-3426.

Sams, M., and Salmelin, R. (1994). Evidence of sharp frequency tuning in the human auditory cortex. *Hearing Research* 75**,** 67-74.

Savastano, M. (2004). Characteristics of Tinnitus : Investigation of over 1400 Patients. 33**,** 248-253.

Scharf, B. (1978). Loudness. *Handbook of perception* 4**,** 187-242.

Schreiner, C.E., and Malone, B.J. (2015). "Representation of loudness in the auditory cortex," in *Handbook of Clinical Neurology*. Elsevier), 73-84.

Schreiner, C.E., Read, H.L., and Sutter, M.L. (2000). Modular Organization of Frequency Integration in Primary Auditory Cortex. *Annu Rev Neurosci* 23**,** 501-529.

Sęk, A., Alcántara, J., Moore, B.C., Kluk, K., and Wicher, A. (2005). Development of a fast method for determining psychophysical tuning curves. *International journal of audiology* 44**,** 408-420.

Sęk, A., and Moore, B.C. (2011). Implementation of a fast method for measuring psychophysical tuning curves. *International journal of audiology* 50**,** 237-242.

Sęk, A., Wicher, A., and Drgas, S. (2007). A fast method for the determination of psychophysical tuning curves: further refining. *Archives of Acoustics* 32**,** 707-728.

Shen, Y., Kern, A.B., and Richards, V.M. (2019). Toward routine assessments of auditory filter shape. *Journal of Speech, Language, and Hearing Research* 62**,** 442-455.

Shen, Y., and Richards, V.M. (2013). Bayesian adaptive estimation of the auditory filter. *The Journal of the Acoustical Society of America* 134**,** 1134-1145.

Shera, C.A., Guinan, J.J., and Oxenham, A.J. (2002). Revised estimates of human cochlear tuning from otoacoustic and behavioral measurements. *Proceedings of the National Academy of Sciences of the United States of America* 99**,** 3318-3323.

Stevens, S. (1957). On the psychopysical law. *Psychol Rev* 64**,** 153-181.

Sutter, M.L. (2000). Shapes and level tolerances of frequency tuning curves in primary auditory cortex: Quantitative measures and population codes. *Journal of Neurophysiology* 84**,** 1012-1025.

Tass, P.A., Adamchic, I., Freund, H.-J., Von Stackelberg, T., and Hauptmann, C. (2012a). Counteracting tinnitus by acoustic coordinated reset neuromodulation. *Restor Neurol Neurosci* 30**,** 137-159.

Trees, D.E., and Turner, C.W. (1986). Spread of masking in normal subjects and in subjects with high-frequency hearing loss. *Audiology* 25**,** 70-83.

Turner, J.J. (1990). "Auditory Dysfunction: Tinnitus," in *Clinical Methods: The History, Physical, and Laboratory Examinations,* eds. Rd, H.K. Walker, W.D. Hall & J.W. Hurst. (Boston).

Tyler, R.S. (2000). *Tinnitus handbook.* United Nations Publications.

Tyler, R.S., and Conrad-Armes, D. (1983). Tinnitus pitch: a comparison of three measurement methods. *British Journal of Audiology* 17**,** 101-107.

Wegel, R., and Lane, C. (1924). The auditory masking of one pure tone by another and its probable relation to the dynamics of the inner ear. *Physical review* 23**,** 266.

Zwicker, E., and Schorn, K. (1978). Psychoacoustical tuning curves in audiology. *Audiology* 17**,** 120-140.
